# Spatial and sectoral water demand assessment for the African continent

**DOI:** 10.1038/s41586-026-11199-8

**Published:** 2026-10-28

**Authors:** Davy Vanham, Dahami Gunathilake, Bhawani Gunasekara, Seifu Tilahun, Youssef Brouziyne, Fasikaw A. Zimale, Monika Rawat, Raphael Vanham, Mohammed El Hafyani, Asma Thowfeek, Sithara Kumarasinghe, S. D. Vaca-Jiménez, P. W. Gerbens-Leenes, Simon Langan

**Affiliations:** 1 International Water Management Institute (IWMI), Colombo, Sri Lanka; 2 International Water Management Institute (IWMI), Accra, Ghana; 3 Bahir Dar University, Bahir Dar, Ethiopia; 4 International Water Management Institute (IWMI), Cairo, Egypt; 5 Institute for Agriculture, Climate and the Environment, University of Southern Queensland, Toowoomba, Queensland Australia; 6 Department of Mathematics, University of Innsbruck, Innsbruck, Austria; 7 Applied Geology and Geo-Environment Laboratory, Faculty of Sciences, Ibnou Zohr University, Agadir, Morocco; 8 Departamento de Ingeniería Mecánica, Escuela Politécnica Nacional, Quito, Ecuador; 9 Integrated Research on Energy, Environment and Society (IREES), University of Groningen, Groningen, The Netherlands

**Keywords:** Hydrology, Water resources

## Abstract

Water demand is the volume of water required by a sector to satisfy its needs^1,2^. Across Africa, these demands are frequently unmet^3^, yet their spatial and sectoral distribution remains incompletely characterized, limiting informed water-management decisions^4^. Here, we provide an Africa-wide estimate of water demand for 2020 across six sectors and multiple subsectors at high spatial resolution. Because the analysis integrates heterogeneous datasets and modelling assumptions, the resulting values should be interpreted as harmonized estimates rather than direct observations of realized water use. We estimate total annual water demand at 347 km^3^, comprising 60 km^3^ (17%) for municipalities (57–64 km^3^, with ±20% key parameters variations), 225 km^3^ (65%) for irrigation (214–233 km^3^, 95% confidence interval, with ±20% variations in irrigation efficiency), 11 km^3^ (3%) for livestock, 15 km^3^ (4%) for manufacturing, 33 km^3^ (10%) for electricity including hydropower and 3 km^3^ (1%) for mining (3–4 km^3^, with annual production variations). Estimated demand increased by 26% between 2010 and 2020. The dominant water-demand sector varies across regions and river basins, broadly reflecting established geographical and socioeconomic patterns. In parts of northern and southern Africa, estimated demand exceeds water availability, although results depend on assumptions regarding environmental-flow protection^5^. Elsewhere, water availability exceeds aggregate demand despite limited access to piped drinking water, indicating that physical availability alone does not determine water security. Our analysis therefore provides a spatially and sectorally resolved baseline for identifying water-demand pressures and access constraints rather than establishing a new causal mechanism of water insecurity. Local estimates should be interpreted cautiously given uncertainties in the underlying datasets.

## Abstract (web summary)

Africa-wide water demand analysis across six sectors in 2020 shows that in specific regions of northern and southern Africa, the current water demand exceeds water availability.

## Main

Africa is a vast continent with heterogeneously spread water availability^6,7^. Yet, how sectoral water use and water demand (WD) are spatially distributed over the continent is not well known. Sectoral WD is the volume of water used by a sector to satisfy its needs, if sufficient water is available^1,2,8^. In many Western countries, sectoral water use generally equals WD; however, in African countries, this is often not the case. As an example, in 2020 only 47% of 1.36 billion Africans had piped drinking water access^3^, mainly because of a lack of adequate infrastructure. The WD of the remaining population that has no piped access is not accounted for in water use statistics of Aquastat^9^.

WD can be expressed in terms of water withdrawal (WW) or water consumption (WC)^10^. WW refers to the volume of water extracted from its source (rivers, lakes, aquifers). WC is the portion of WW that is not returned to the original water source after being withdrawn. Water use is a generic term that can mean both.

Sectoral water uses are difficult to quantify, making related data hard to find. The only way to accurately quantify real-world physical water uses is by measuring them, yet such data are only scarcely available.

A persistent lack of reliable water data and information is a widespread constraint on water management in the Global South^4^. For detailed water management assessments and informed policy decisions, knowing detailed WDs of all sectors in high spatial resolution is a necessity^10^. It is also important for researchers and stakeholders working in Africa to obtain open-access data, as they often lack the funds to purchase high-quality data. There is thus an urgent need for open-access, reliable, spatially distributed information on WDs covering Africa, including all relevant sectors. This limits research efforts and resulting science-based decisions among stakeholders.

To fill this data gap, we conducted an Africa-wide year 2020 WD analysis, at unprecedented spatial resolution (5 arcmin for 2 sectors, 2.5 arcminutes (arcmin) for 2 sectors and point data for 2 sectors) as well as unprecedented sectoral and subsectoral detail. The spatial resolutions of 2.5 arcmin and 5 arcmin equal about 4.5 km and 9 km at the equator, respectively. We quantify WD in terms of WW. We account for six sectors. The first sector is municipal WD (piped and non-piped population) at 2.5 arcmin resolution. We use actual water supply and water loss data of more than 450 water supply utilities spread over Africa to assess piped WD. These utilities are primarily sourced from the International Benchmarking Network (IBNET; https://newibnet.org), a unique information source for utility-specific water data. This is a first-time novelty, to our knowledge, that no existing study covering Africa^11,12^ has done.

The second sector is irrigation WD for 46 Spatial Production Allocation Model (SPAM) crops and major crop groupings^13^. The third sector is livestock WD (buffaloes, cattle, chickens, goats, pigs and sheep). The fourth sector is the manufacturing industry WD, in 2.5 arcmin resolution, differentiating between several subsectors such as ‘food and beverages’, ‘clothing’ or ‘paper and paper products’. The fifth sector is electricity production at 2,534 individual power plants, for 10 different fuel types, that is, the fossil fuels coal, oil and gas, the renewables hydropower, solar, wind, biomass and geothermal as well as two other fuel types (nuclear and waste heat). The sixth sector is mining WD, for 682 individual mines, differentiating between 34 main commodities such as gold, diamonds or iron ore.

Africa is developing fast because of population increase, economic growth, urbanization and shifting consumption patterns. These developments have profound effects on sectoral WDs. To analyse the change in sectoral and subsectoral WDs for the whole of Africa, we conduct our analysis by using the same input data and methodologies for the year 2010 and compare the results with the year 2020. The current lack of temporal WD data has led to marked gaps in knowledge of water-use trends across different sectors throughout Africa.

None of the existing global water use and WD studies^11,12^ have reached the level of sector and subsectoral and spatial detail, or the explicit focus on Africa, that we provide here. We provide open access to all our results.

Furthermore, we assess how WD relates to water availability, accounting for environmental flows (EFs). The latter are minimum flows required to maintain ecosystem integrity in rivers and other water bodies^5,14^. We use two EF methods, one accounting for high EF protection, the presumptive standard in ref. ^15^. The other method is the variable monthly flow (VMF) method^16^, accounting for lower EF protection. We thereby analyse whether Africa has the water resources to provide current WD and identify river basins in which WDs are high relative to available water availability.

Our study sheds light on the temporal evolution of sectoral WDs in Africa from 2010 to 2020, and on the question of whether Africa suffers from water scarcity and/or a water management problem.

## Sectoral WD in year 2020

**Fig. 1:**
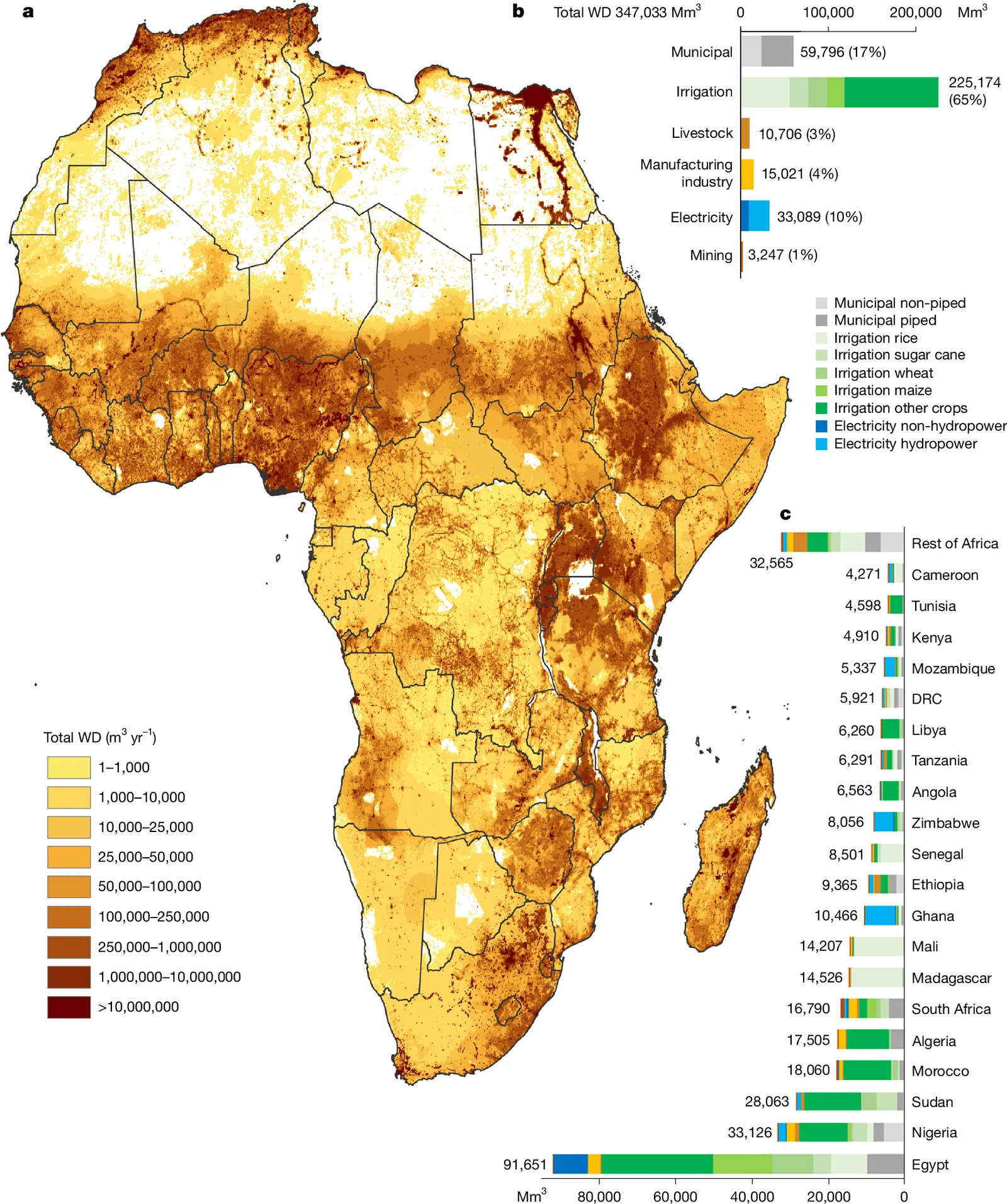
Total WD for Africa in 2020. **a**, Spatial distribution of total WD in m^3^ yr^−1^. **b**, WD of main sectors and subsectors in Mm^3^. **c**, WD (Mm^3^) of main sectors and subsectors at the national level, with the 20 largest WD countries shown in decreasing order (Egypt to Cameroon). DRC, Democratic Republic of the Congo.

Total WD is heterogeneously spread over Africa, amounting to 347,033 Mm^3^ (347 km^3^) (Fig. 1 and Supplementary Data 1 and 2). Municipal WD accounts for 59,796 Mm^3^ (17%), irrigation for 225,174 Mm^3^ (65%), livestock for 10,706 Mm^3^ (3%), manufacturing industry for 15,021 Mm^3^ (4%), electricity including hydropower for 33,089 Mm^3^ (10%) and mining for 3,247 Mm^3^ (1%).

Municipal WD accounts for people with piped access (35,807 Mm^3^) and non-piped access (23,989 Mm^3^). For irrigation, the main crops are rice, sugarcane, wheat and maize. For electricity, hydropower accounts for 23,035 Mm^3^, whereas other fuel types account for 10,053 Mm^3^.

Countries with high populations, and/or irrigated areas and/or high electricity WDs, account for the largest WD amounts (Fig. 1 and Supplementary Data 1). Egypt has the highest WD, followed by Nigeria, Sudan and Morocco (Fig. 1c).

**Fig. 2:**
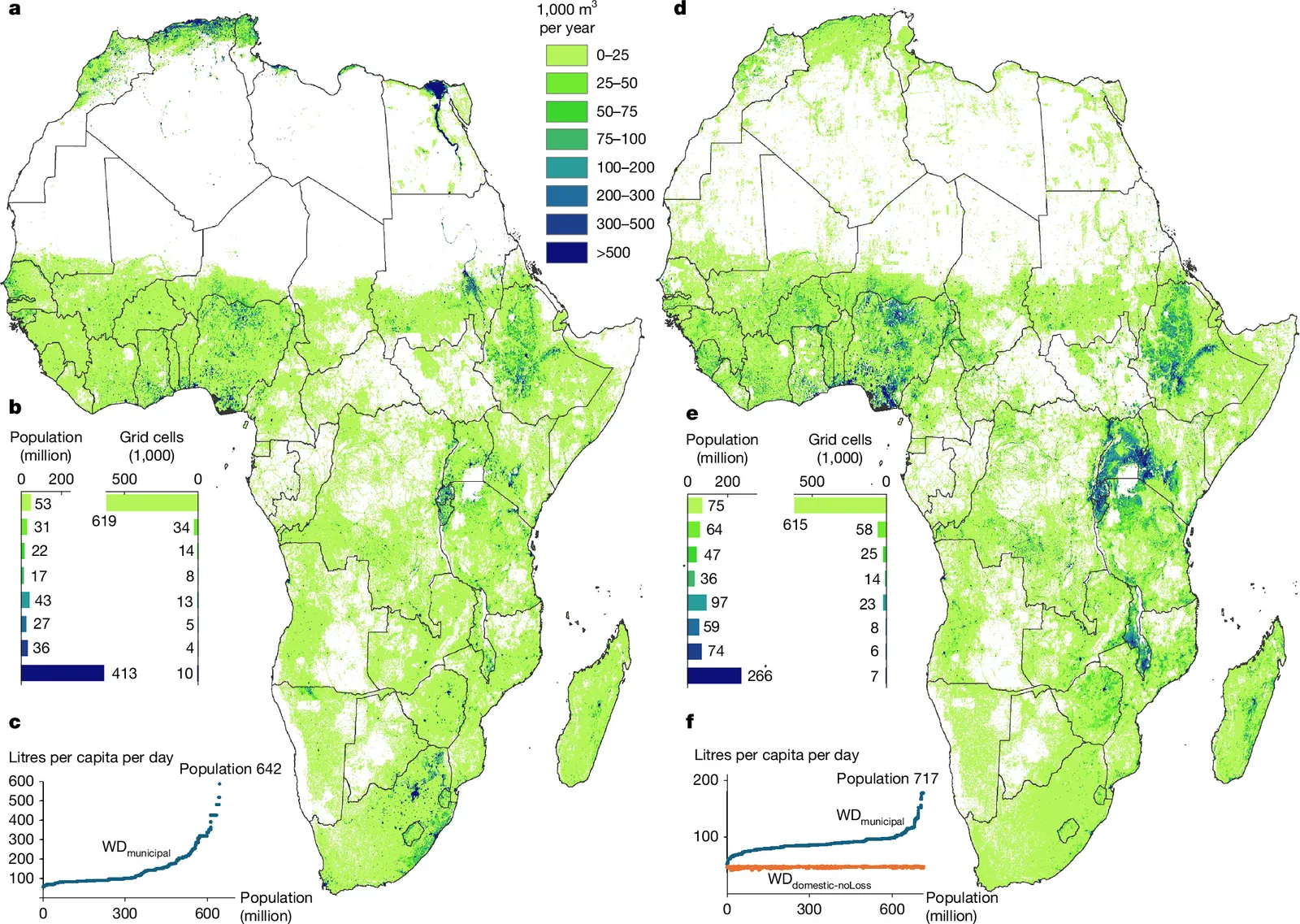
Municipal WD for Africa in 2020. **a**–**c**, Piped water access WD. Spatial distribution of WD (1,000 m^3^ yr^−1^ in different grid cell classes) (**a**); population (million) and number of grid cells (1,000) for the different grid cell classes (**b**); relationship between population (million) and per capita WD_municipal_ (l cap^−1^ day^−1^) (**c**). **d**–**f**, Non-piped water access WD. Spatial distribution of WD (1,000 m^3^ yr^−1^ in different grid cell classes) (**d**); population (million) and amount of grid cells (1,000) for the different grid cell classes (**e**); relation between population (million) and per capita WD_municipal_ (l cap^−1^ day^−1^), calculated WD _domestic-noLoss_ according to equation (2) (**f**).

In total, 642 million Africans have piped water access (35,807 Mm^3^) (Fig. 2a and Supplementary Data 1, 2 and 4), whereas 717 million Africans lack piped water access (23,989 Mm^3^) (Fig. 2d and Supplementary Data 1, 2 and 4). Piped water access WDs are spread over Africa, with high amounts concentrated in countries in which the access rate is high, such as in many northern African countries (Egypt 99%, Morocco 78% and Tunisia 92%), southern African countries (South Africa 90%, Botswana 91% and Namibia 83%) and selected western African countries (Senegal 75%, Gabon 88% and Gambia 78%). Within countries, piped access is concentrated in urbanized areas^17^. We calculate 413 million people in less than 10,000 urbanized grid cells, whereas about 619,000 sparsely inhabited grid cells represent only 53 million people (Fig. 2b).

Actual water supply and water loss data of more than 450 water supply companies were used to quantify piped water access WD. Whereas per capita WD for 294 million Africans (46% of population with piped access) is 100 litres per person per day (l person^−1^ day^−1^) or less, for 54% this amount is exceeded (Fig. 2c). For 201 million Africans (31%), per capita WD is from 100 l person^−1^ day^−1^ to 200 l person^−1^ day^−1^, for 68 million Africans (11%) it is between 200 and 300 l  person^−1^ day^−1^ and for 78 million Africans (12%) it exceeds 300 l person^−1^ day^−1^. High per capita WD amounts are concentrated in northern and southern African cities, such as the Windhoek municipality utility (244 l person^−1^ day^−1^), the Damietta Water and wastewater Company in Egypt (315 l person^−1^ day^−1^), Cape town metro utility (254 l person^−1^ day^−1^) or SEAAL (Société des Eaux et de l’Assainissement d’Alger) (249 l person^−1^ day^−1^).

Non-piped water access WDs are spread over Africa, with high amounts concentrated in countries in which the access rate is low, generally in West, Central and East Africa (Fig. 2d). Of 717 million Africans who do not have piped access, we calculate that 608 million (85%) have a per capita WD of 52–100 (Fig. 2f).

**Fig. 3:**
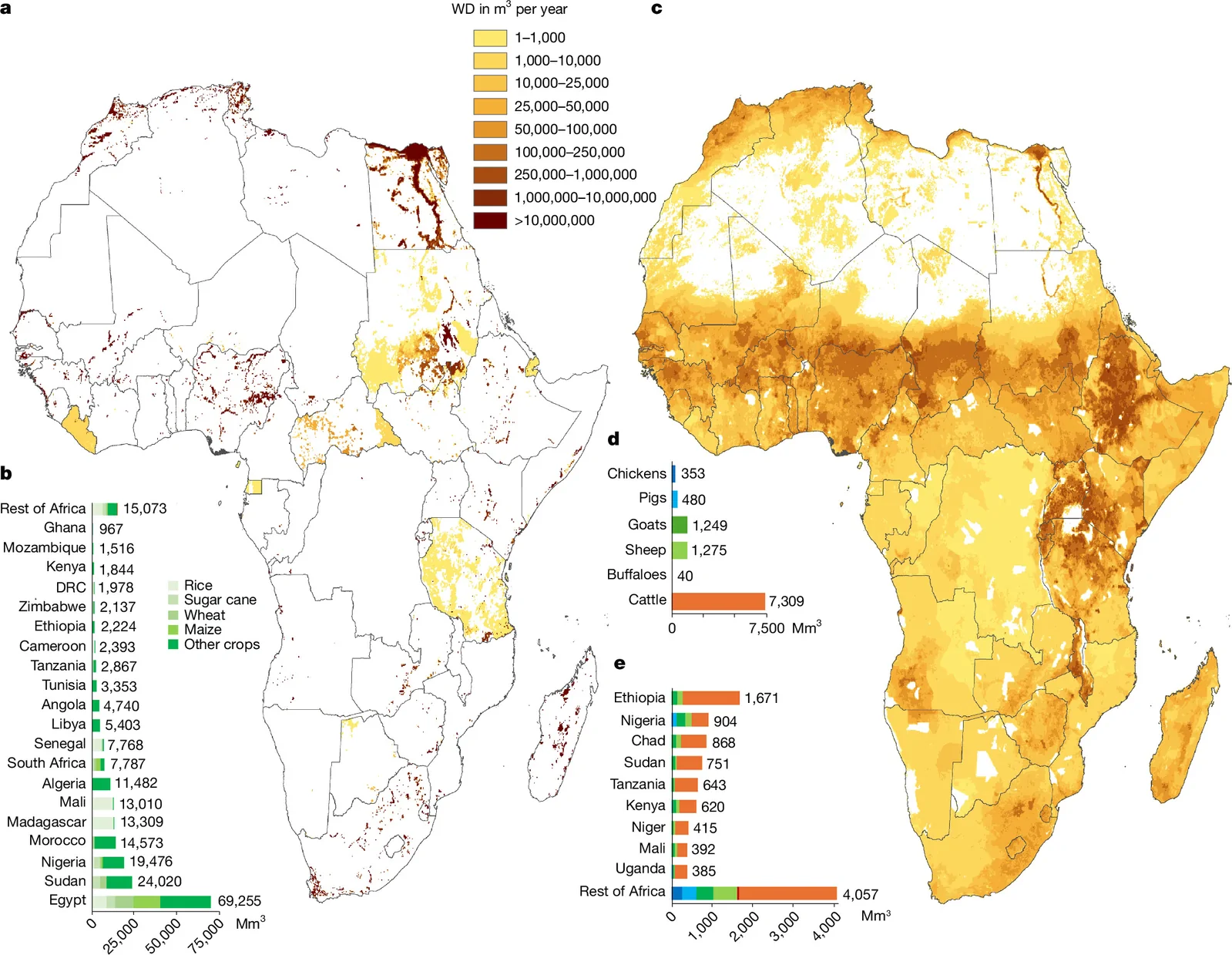
Agricultural WD for Africa in 2020. **a**, Spatial distribution of irrigation WD (m^3^ yr^−1^). **b**, National irrigation WD (Mm^3^ yr^−1^) with differentiation between rice, sugar cane, wheat, maize and other crops. **c**, Spatial distribution of livestock WD (m^3^ yr^−1^). **d**, Continental WD (Mm^3^ yr^−1^) for six livestock classes. **e**, National livestock WD (Mm^3^ yr^−1^) with differentiation between livestock classes.

Irrigation WDs are heterogeneously spread over Africa, with the highest national amounts located in Egypt, Sudan and Nigeria.(Fig. 3a,b and Supplementary Data 1). Rice, sugar cane, wheat and maize account for the largest WD amounts. Among remaining crops, tropical fruits, temperate fruits, vegetables and groundnuts have large WDs.

Livestock WDs are concentrated in East Africa and the Sahel, with the highest national amounts in Ethiopia, Nigeria, Chad and Sudan (Fig. 3c–e). Cattle account for the bulk of this WD, followed by sheep, goats, pigs, chickens and buffaloes. These amounts are dependent on the size of the livestock classes and their unit drinking and service WD. The chicken population in Africa was approximately 2.2 billion heads, making chickens the most numerous livestock. Their unit WD is small when compared with cattle with 371 million heads.

**Fig. 4:**
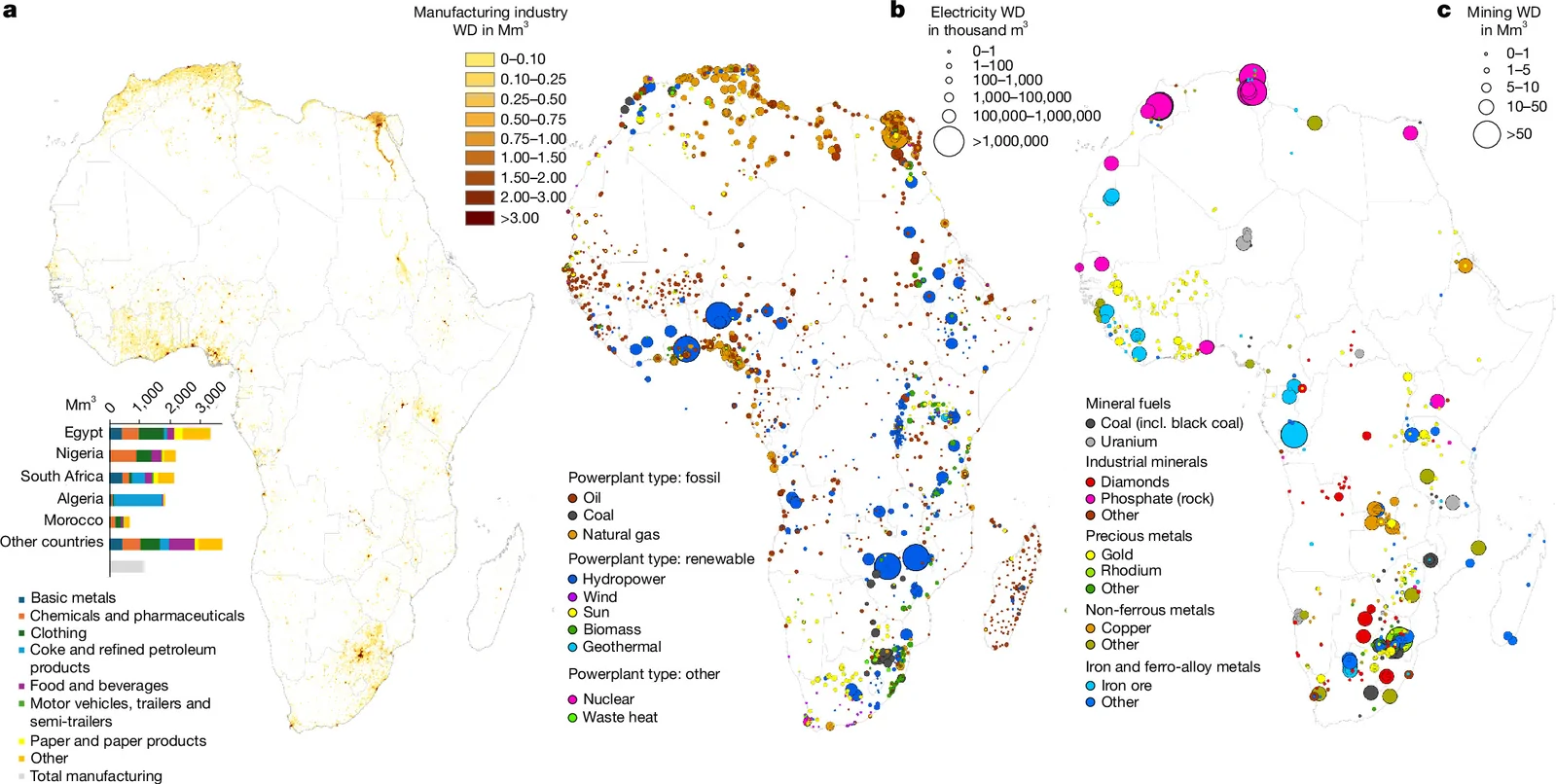
Industrial WD for Africa in 2020. **a**, Spatial distribution of the manufacturing industry (Mm^3^ yr^−1^) with subsectoral display largest national amounts. **b**, Spatial distribution of electricity (1,000 m^3^ yr^−1^) according to fuel types (the size of the circle relates to amount). **c**, Spatial distribution of mining (Mm^3^ yr^−1^) according to main commodities (the size of the circle relates to the amount).

Industrial water use in Aquastat includes the manufacturing industry, electricity production (cooling of thermoelectric plants) and mining. We quantify these three sectors separately, including different subsectors (Fig. 4 and Supplementary Data 6). The WD of the manufacturing industry is heterogeneously spread over Africa, with the highest national amounts located in Egypt, Nigeria, South Africa and Algeria (Fig. 4a). The manufacturing sector is diverse, it includes different subsectors with varying degrees of water intensity. Our quantification is based on data on subsectoral gross value added (GVA) for the manufacturing industry^18^ and its water intensity. Some countries do not provide subsectoral GVA data. For these we quantified a total manufacturing industry WD of 1,150 Mm^3^.

The WD for electricity is based on 2,534 individual power plants, including 1,447 fossil, 1,071 renewable and 16 other power plants (Fig. 4b). With 23,035 Mm^3^, hydropower is the largest WD component in this sector, with the largest proportion located in sub-Saharan Africa. The other fuel types account for 10,053 Mm^3^. Fossil fuels account for 504 Mm^3^ (oil), 477 Mm^3^ (coal, especially in southern Africa) and 9,002 Mm^3^ (natural gas, especially in North Africa). The other renewables account for 13 Mm^3^ (solar), 49 Mm^3^ (biomass), 0 Mm^3^ (wind) and 2 Mm^3^ (geothermal, in the Great Rift Valley of Kenya). Nuclear power WD accounts for 5 Mm^3^ (the Koeberg nuclear power plant in the Republic of South Africa (RSA)) and waste heat for 1 Mm^3^.

The mining WD is based on 682 individual mines (Fig. 4c and Supplementary Data 5). Large mining WDs are located in southern Africa. Coal accounts for 423 Mm^3^ and uranium for 107 Mm^3^. Diamonds account for 114 Mm^3^ and phosphate (rock) for 1,059 Mm^3^, with the largest amounts in Morocco and Tunisia.

**Fig. 5:**
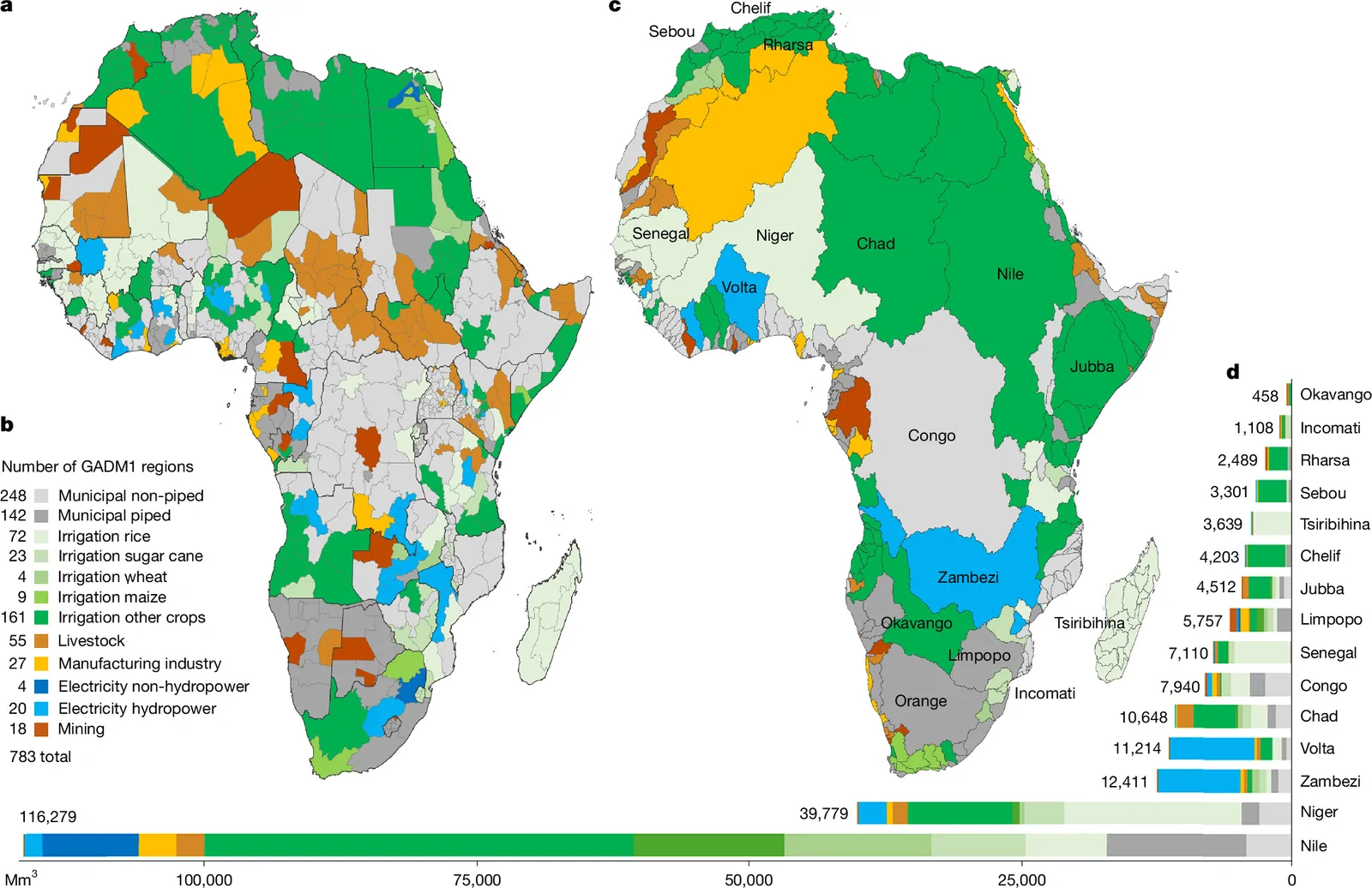
Principal sectoral and subsectoral WD within subnational regions as well as main river basins. In total,12 sectors and subsectors are used. **a**, Principal sectoral and subsectoral WD within GADM level 1 subregions. **b**, Number of GADM1 regions for a principal WD of 783 regions. **c**, Principal sectoral and subsectoral WD within main river basins. **d**, WD amounts (Mm^3^ yr^−1^) for main river basins.

Which sector or subsector accounts for the highest WD varies heterogeneously across regions and river basins (Fig. 5). The subnational level shows a wide variety among 12 sector and subsector principal WDs (Fig. 5a,b). For a total of 783 subnational regions, all 12 sectors and subsectors are represented, from a minimum of four regions (for irrigation wheat and electricity non-hydropower) to a maximum of 248 regions (for municipal non-piped). The latter shows the importance of including non-piped municipal WD. Mining, which represents only 1% of African total WD, is the principal WD in 18 regions. This is, for example, the case in the Erongo region of Namibia, with its large uranium mines, or in the Ganzhi and Southern regions of Botswana, with their large diamond mines. Neglecting a certain sectoral WD can thus have a large effect on the total WD, especially locally.

River basins also show wide variety in their principal sectoral and subsectoral WD (Fig. 5c,d and Supplementary Data 1). In large river basins such as the Nile and the Niger, the principal WD is the irrigation of other crops and rice, respectively. In the Zambezi and Volta, however, it is electricity from hydropower. In the Congo basin, it is non-piped municipal WD. In the Orange and Limpopo basins, it is piped municipal WD.

## WD related to water availability

**Fig. 6:**
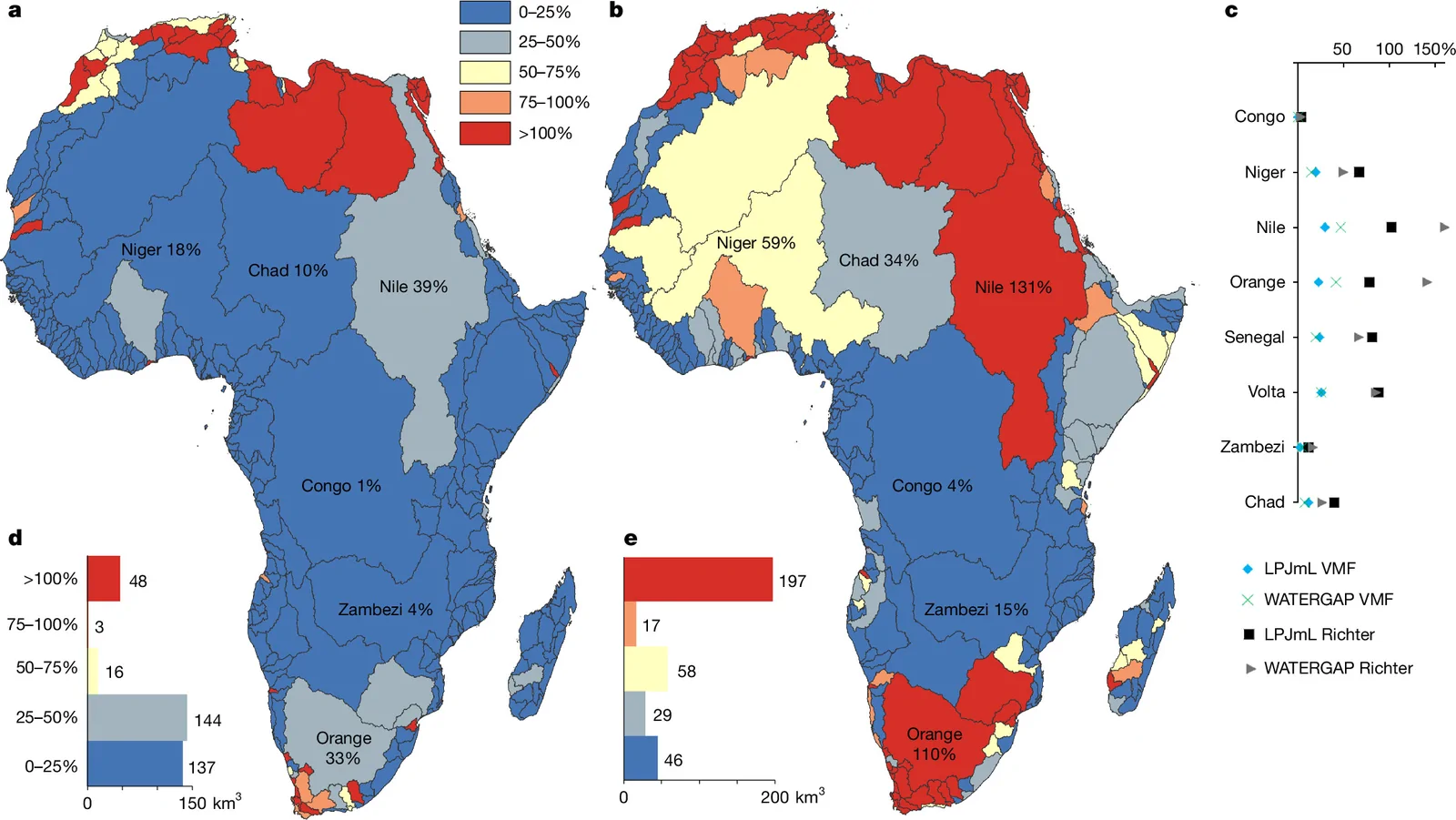
WD related to available renewable water resources in African river basins. Amounts in percentage for different classes: 0–25%, 25–50%, 50–75%, 75–100% and exceeding 100%. **a**, The average of LPJmL and WATERGAP with the VMF method. **b**, The average of LPJmL and WATERGAP with the Richter method. **c**, The individual hydrological model and EF method for selected main river basins. **d**, Total WD (km^3^) in different classes for **a**. **e**, Total WD (km^3^) in different classes for **b**.

Total WD related to water availability with lower EF protection (the VMF method), results in values up to 100% in most river basins throughout Africa (Fig. 6a). When values are below 100%, EFs are not violated. These basins include the Nile, Chad, Niger, Congo and Zambezi. These basins have enough annual renewable water availability to provide total WD. For some river basins in North and southern Africa however, values exceed 100. This means that theoretically EFs are violated. These river basins represent a WD of 48 km^3^ (Fig. 6d, the VMF method), 14% of the total WD of 347 km^3^. They include small basins in the Western Cape province of South Africa, a region well-known for water scarcity^19^. They also include the Chelif and Hodna basins in Algeria, the Rharsa basin in Algeria and Tunisia and many regions along the Mediterranean coast.

For the Richter EF method, the total WD related to water availability results in values exceeding 100% in more river basins throughout Africa (Fig. 6b). Many basins in North Africa, including the Nile, as well as several basins in southern Africa, including the Orange and the Limpopo basins, show amounts higher than 100%. Combined, these basins represent 197 km^3^ (Fig. 6e, Richter method), 57% of the total WD of 347 km^3^. It is therefore questionable whether this high level of EF protection can be maintained in these basins. However, this high level can be maintained in many other basins, such as the Congo (Fig. 6b), the Zambezi, Chad or Niger.

Trade-offs between human WD and the level of EF protection are important in many African river basins. These considerations should include the different ecosystem services EFs provide for humans, many with direct links to specific Sustainable Development Goals (SDGs)^10,20^. As an example, EFs sustain crocodiles and hippos in basins with wildlife tourism, which brings revenue for local communities up to the national level^21^. These basins include the Limpopo, flowing through the Kruger National Park, or the Tana River basin in Kenya. Careful consideration of trade-offs between human WDs and the level of EF protection in these basins can be made by evaluating more site-specific EFs^22^.

## WD increased from 2010 to 2020

Overall, the WD of all sectors in Africa has increased from 274,763 Mm^3^ to 347,033 Mm^3^ (by 72,270 Mm^3^ or 26%), for the period from 2010 to 2020 (Extended Data Table 1).

Irrigation WD increased 25%, driven by population increase, economic growth, changing diets and consumer preferences^23,24^. This has led to farmers aiming to increase production and close water-limited crop yield gaps by expansion of irrigated areas^13,25^, resulting in substantially higher WDs for crops such as rice, maize, fruit, vegetables, groundnuts, soybeans and sugar cane (Supplementary Table 3).

Although African diets have historically relied on staple crops such as maize, sorghum and cassava, there has been a shift towards preferences in rice consumption, especially in western Africa and Madagascar, where per capita consumption now exceeds 100 kg annually^24,26^. An increase in Africa-wide rice consumption from 32 to 44 million tonnes from 2010 to 2020 has been met through a parallel increase in rice domestic production (60% of consumption) and imports (40% of consumption) from primarily Asia^24^. As a result, rice WD has increased by 38% from 39,999 Mm^3^, mostly in countries such as Senegal, Madagascar, Mali, Mauritania, Nigeria and Cameroon. Increases in irrigated rice areas are associated with exposure to greater malaria risk^27^, yet alternate wetting and drying in African rice production has proven to decrease this exposure and reduce the WD of rice fields^28^.

Large parts of the population in African countries do not eat recommended amounts of fruit and vegetables, depriving them of essential nutrients^24,29–31^. African farmers have increased domestic fruit and vegetable production^24^, resulting in a WD increase by 24% from 28,392 Mm^3^ for fruits and by 115% from 9,006 Mm^3^ for vegetables. This increased production has led to more overall fruit and vegetable consumption throughout Africa^24^. A substantial part has also been used for export, resulting in so-called virtual water export, both inside (between African nations) and outside the continent^32^. This export includes bananas from Côte d’Ivoire and Cameroon, citrus fruit and grapes from South Africa and northern Africa^24^, or tomatoes from Morocco, often contributing to local water stress (Fig. 6).

The double burden of malnutrition, that is, the coexistence of undernutrition (stunting and wasting) alongside overnutrition (overweight and obesity), is prevalent throughout Africa^29^. In many regions and socioeconomic classes, the per capita intake of animal-sourced foods is among the lowest globally^24,29,33,34^. Some nations have therefore developed policies to increase the production and consumption of animal-sourced foods to address nutrient gaps, especially for eggs and dairy^35,36^. Alongside population increase, rising income and urbanization, these factors have driven the expansion of livestock numbers throughout Africa^37,38^. Consequently, from 2010 to 2020, we quantify large Africa-wide WD increases for cattle (+30%), goats (+48%), sheep (+26%) and chickens (+29%) (Extended Data Table 2 and Supplementary Table 5), with large increases in Chad, Ethiopia, Tanzania and Nigeria.

Municipal WD increased 29% (Extended Data Table 1), driven by population increase (+30% or 305 million additional people) as well as the efforts of the countries to increase WASH for their populations^3^. In Egypt, for example, piped WD increased by 2,131 Mm^3^ (+27%), with a population increase of 23% and an increase in connection rate from 95% to 99%, in line with Vision 2030 of Egypt. The RSA increased access to piped water supply from 11.5 to 14.6 million households^39^ for a growing population, resulting in a piped WD increase of 753 Mm^3^ (+22%). Ethiopia has been and is actively working to increase household piped water connections through the multi-sector ‘One WASH National Program’ launched in 2013, increasing access rates from 28% to 43% for a growing population.

**Extended Data Fig. 1:**
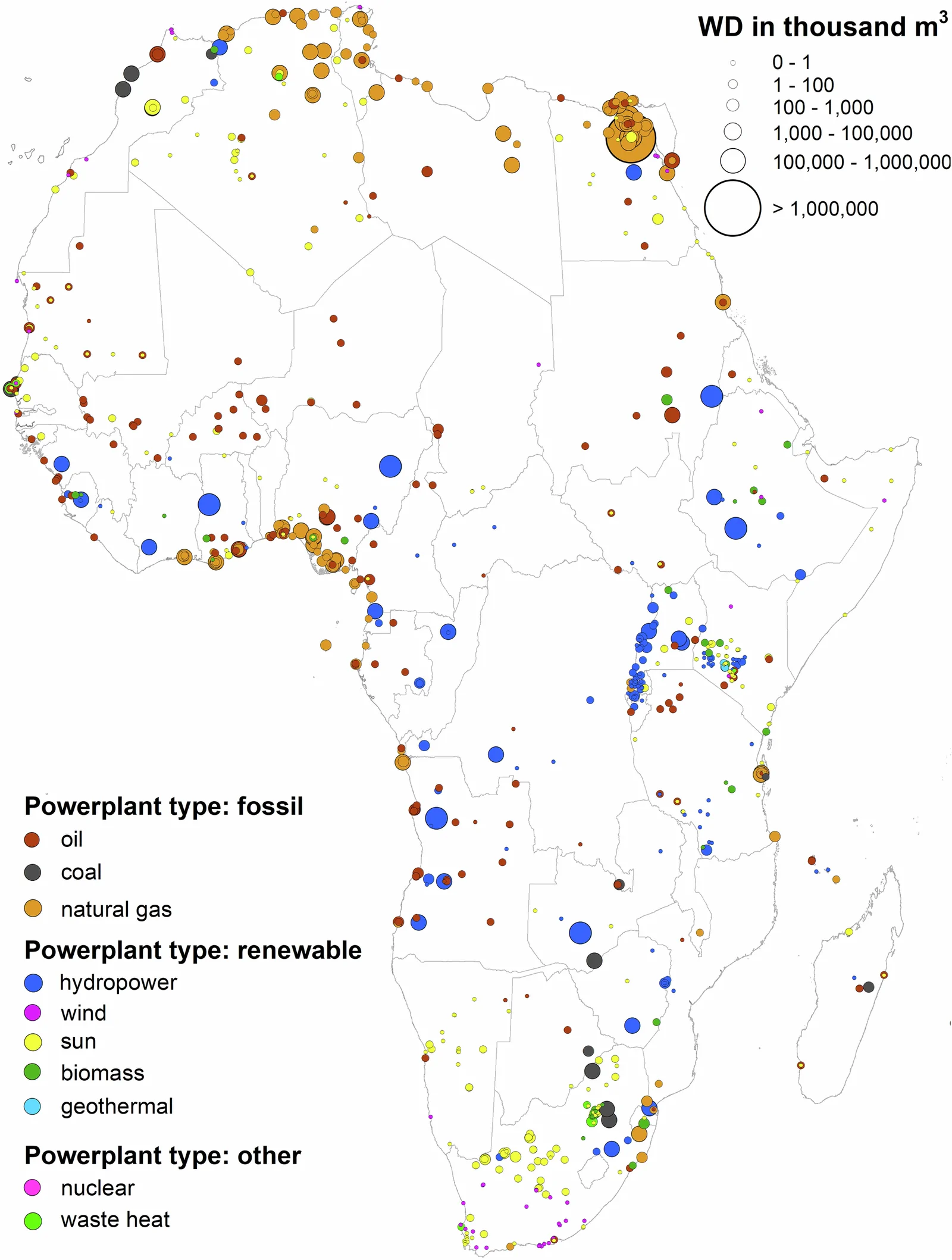
Additional WD electricity from 2010 to 2020, according to fuel types. Spatial distribution of additional electricity WD (1,000 m^3^ yr^−1^) that became operational from 2010 to 2020, according to fuel types (the size of the circle relates to amount).

Africa is still experiencing a persistent, multi-faceted electricity gap, with more than 500 million Africans without access to electricity in 2020. Power plant expansion from 2010 to 2020 has increased electricity generation and access throughout Africa. We identify 846 additional power plants that became operational from 2010 until 2020, that is, from 1,511 to 2,357 operational power plants, almost doubling electricity production to 1,046,061 GWh (Extended Data Fig. 1). Electricity WD increased 24% (Extended Data Table 3 and Extended Data Fig. 1). Particularly, the WD related to gas-fired power plants increased (104%) because of expansion of such plants in primarily northern Africa and Nigeria. Hydropower, a water-intensive electricity source, expanded less (+7% in WD) compared with gas-fired plants. To decarbonize the continent, many African countries invested in solar and wind expansion, energy sources that have a low water intensity in terms of WD per electricity unit produced^40^, only adding a WD of 13 Mm^3^ while adding production of 19,801 GWh.

The WD of the manufacturing industry increased Africa-wide with 3,161 Mm^3^ (+27%), albeit with strong regional differences (Extended Data Tables 1 and 4 and Supplementary Data 6). Throughout Africa, the manufacturing industry contributes only a small part to GDP. However, in some countries such as Egypt or South Africa, WDs have increased and constitute a substantial part of the total national WD. In some countries, we computed a decrease in WD, such as Algeria, which passed its peak crude oil production around 2010, with output and related WD decreasing up to 2020. The clothing subsector shows a substantial increase in WD (from 1,301 Mm^3^ to 2,290 Mm^3^), with large increases in Egypt, which is emerging as a global player in textiles and apparel production. Moreover, the food and beverages industry shows a large WD increase because of increased demand for processed foods and beverages.

The WD of mining increases substantially by 65%, from 1,964 Mm^3^ to 3,247 Mm^3^, the result of new mines and higher production in existing mines for different commodities. Increased phosphate rock mining in Morocco is driven by increased demand from global agriculture^41^, resulting in a WD increase of 257 Mm^3^. Morocco holds more than 70% of global phosphate rock reserves^42^. In the RSA, the WD for coal increases by 230 Mm^3^, driven by growing electricity demands from coal-fired power plants. Also the WDs of iron ore, gold, diamond, cobalt, copper, rare earth and uranium increase substantially. The WD of cobalt mining increases almost 500%, especially in the Democratic Republic of the Congo, driven by soaring cobalt demands worldwide, such as for producing electric vehicle batteries^43^.

## Discussion

Our Africa-wide WD analysis in unprecedented spatial and sectoral and subsectoral detail, shows a heterogeneously spread spatial pattern over the African continent and a substantial increase in sectoral WDs from 2010 to 2020. Our results stress the importance of accounting for the WD of all different sectors and subsectors in great spatial detail, as any sector or subsector can represent the highest WD in a region or river basin (Fig. 5). All sectoral WDs are required to compute water stress (Fig. 6). Omitting certain sectors or using too coarse spatial resolutions can lead to biased recommendations and decision-making.

Combined with available water resources, which are also not evenly distributed, this results in a diverse spread of river basins, where at present WD exceeds available water resources or not, depending on trade-offs in EF protection. Water scarcity is now concentrated in the dry areas of North Africa and southern Africa. Nevertheless, WDs are often not met Africa-wide, even in wetter regions, because of a lack of adequate infrastructure. Non-piped water access is lacking for 717 people with a total WD of 23,989 Mm^3^, much of whom are located in basins that are not water stressed. Massive investments are needed to increase water access and to meet different SDG 6 targets by 2030. Africa, therefore, shows both water scarcity and water management problems.

Our results should be regarded as best estimates and not as absolute amounts. They are the result of a combination of intensive data mining, explicitly looking for as much African-specific data as possible, modelling and spatial analyses, which include different assumptions and input data gaps. Inherent to any modelling study, this leads to uncertainty in the WD amounts we present. We therefore validate our results through different means and conduct selected uncertainty and sensitivity analyses, as discussed in detail in Supplementary Information sections 3 and 4. In the former, we validate our results by (1) comparing them with national data from Aquastat^9^ (Supplementary Table 5); (2) comparing them with the few global studies that provide spatially distributed sectoral WD data covering Africa (Supplementary Tables 6–9 and Supplementary Figs. 1–4), and (3) comparing them with other modelling approaches (Supplementary Table 10 and Supplementary Fig. 5). We show the novelty in comprehensiveness as well as groundbreaking spatial and sectoral and subsectoral resolution of our study, compared with existing studies.

In Supplementary Information section 4, we show robustness in our results by ±20% variations in key parameters for analysing irrigation WDs (Supplementary Table 11 and Supplementary Fig. 6), municipal WD (Supplementary Table 12) and mining WD (Supplementary Table 13) for the year 2020. For irrigation WDs, for example, ±20% variations in irrigation efficiency put our total amount of 225,174 Mm^3^ in a 95% confidence interval of 213,736–232,818 Mm^3^. Variations of ±20% for the factors physical water loss, as well as the proportion of domestic water use within municipal water use, result in the total municipal WD varying between −-9 and +13%. Limitations in this study include the reliance on some non-regional industrial water intensity data, for which reliable African data could not be found, or the fact that night-time light intensity maps can overlook rural informal sectors when spatially distributing national industrial WDs.

Africa is rapidly growing and changing, with WDs projected to increase substantially in the future^44,45^, because of population increase, shifting lifestyles and growing economies. Our 2020 and 2010 WD analyses can be considered as a baseline, from which there is a need to analyse future growing sectoral and subsectoral WDs with high spatial resolution.

Future sustainable development should take current sectoral WDs and resulting local water stress into account. Recommendations and policy implications include (1) the increase of water efficiencies and reduction of water losses for all sectors and subsectors; (2) the application of integrated water resources management (IWRM) with a nexus approach, while accounting for available water resources and EFs; (3) the development of alternative water resources such as water recycling or desalination; (4) massive investment to provide WASH to 100% of the African population; and (5) evaluation of the costs and benefits of agricultural and related virtual water exports outside Africa.

Water losses, high in many African countries for sectors such as municipalities and irrigation, need to be reduced. By including actual water loss data from water supply companies, we confirm high average physical water losses in African water supply networks. These range from low values of 10% in Windhoek, a water-scarce city, in which much water infrastructure investment has been done^46^, more than 19% in Cape Town and 27% in Johannesburg to values exceeding 40% and even 50% in different Nigerian, Kenyan and Ethiopian utilities. Reducing physical water losses would decrease municipal WDs substantially.

IWRM at the river basin, national and subnational levels should be implemented, accounting for the WD of all sectors and water availability, addressing trade-offs and win-wins. This requires coordination and collaboration between different ministries and stakeholders, aligning policies on water, agriculture, energy, industry and the environment. Also transboundary cooperation is required, as many African river basins are international (Fig. 6). This also includes the evaluation of where and how additional WDs can be further developed, addressing water resilience in investment strategies. As an example, future power plant planning and development needs to account for water and carbon intensities of different fuel types^45^. Hydropower is vulnerable to drought, requiring a mix of energy source investments. Further expansion of the low-cost energy sources such as solar and wind is a win-win in many African settings. Another example is the expansion of rice production, which should focus on areas without water stress and WD reductions by alternate wetting and drying^28^.

To reduce water stress, alternative water resources such as water recycling or desalination need to be developed. These water management practices are already practised in water-scarce regions such as Northern Africa^47^. WD management is crucial for balancing water availability and demand, particularly in areas facing water scarcity or growing WD pressures.

The fourth recommendation is to massively invest in the expansion, rehabilitation and maintenance of water supply for all Africans as well as the collection and purification of wastewater. The fifth recommendation is to evaluate the benefits (revenue) and costs (national food security, depletion of water resources) of the (virtual water) export related to (cash) crops from different African countries outside the continent, especially from water-scarce areas.

## Methods

### General

We assess the sectoral and subsectoral WD for the years 2020 and 2010, for municipal WD, irrigation, livestock, the manufacturing industry, electricity production and mining.

WD is the volume of water used by a sector to satisfy its needs, if sufficient water is available^1,2,8^. When sufficient water is not available, the need is not met, often leading to underperformance of a specific activity. As an example, during periods of low water availability such as dry periods, farmers might not have sufficient water to irrigate the full WD of their crops, leading to lower crop yields. Another example is the lack of drinking water infrastructure, so that a person has to fetch water from a distant water source. This person can thereby be deprived of a minimum water need for activities such as washing or cleaning. In our assessment, we quantify WDs to satisfy all sectoral needs.

We quantify WD in the sense of WW, not WC^10^. Many different definitions of water use exist, sometimes leading to confusion when they are not properly defined and applied. WW, also called water abstraction, refers to the volume of water withdrawn or extracted from its source (rivers, lakes and aquifers). WC refers to the portion of WW that is not returned to the original water source after being withdrawn, such as through evaporation. WW minus WC amounts to the return flow. Water use is a generic term that can apply to either WW or WC.

We compute all sectors in a spatial resolution of 5 arcmin (which accounts for 371,665 data points), apart from municipal and industrial WD, which we compute at 2.5 arcmin spatial resolution.

The spatial resolution we use is 2.5 arcmin, which equals 0.041667° or about 4.5 km at the equator. For each grid cell of 0.5°, the resolution of most existing studies, we quantify 144 grid cells. We also use 5 arcmin, which equals 0.08333° or about 9 km from the equator. For each grid cell of 0.5°, the resolution of most existing studies, we quantify 36 grid cells. We use point data for mining and electricity, the spatially most detailed resolution possible. For the total WD (the sum of all six sectors), the restricting sectors are irrigation and livestock, as their input data (SPAM crops^13^ and Gridded Livestock of the World or GLW data^48,49^, respectively) are available in 5 arcmin. No crop- or livestock-specific spatially distributed data covering Africa are available at higher spatial resolution. The spatial resolution 5 arcmin for total WD is therefore the highest resolution possible.

The African population in 2020 was 1,36 billion people, whereas in 2010, it was 1,05 billion people (an increase of 30%).

We include mainland Africa and Madagascar, which totals 49 countries (Morocco, including Western Sahara). We do not include the following small islands: Cabo Verde, Comoros, Mauritius, Mayotte, Réunion, Saint Helena, Ascension and Tris, São Tomé and Príncipe, Seychelles.

**Extended Data Fig. 2:**
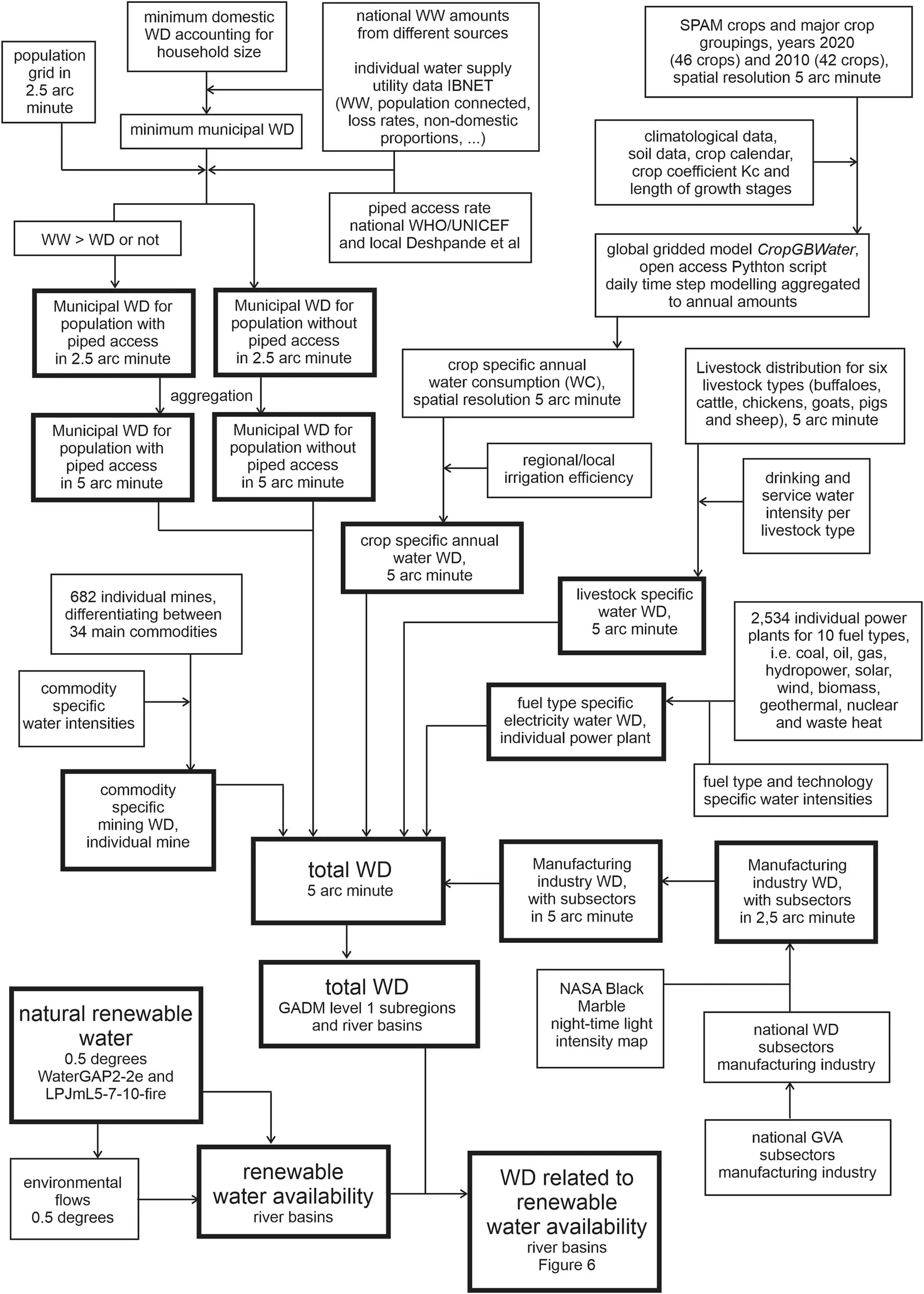
Workflow methodology. Visual presentation of the methodology workflow. The methodology can be defined as a bottom-up approach.

Our assessment is based on an intensive data mining campaign, modelling and GIS analyses. Supplementary Table 1 provides an overview of the main data sources used in this study, whereas many additional sources are used, including those listed in Supplementary Table 2. We present a general methodology workflow as Extended Data Fig. 2. The methodology can be defined as a bottom-up approach.

All maps are created with ArcGIS using country/administrative borders from the database of Global Administrative Areas (GADM)^50^. All figures are produced with the ‘Equal Earth projection’^51^, which is endorsed by the African Union^52^ and recommended in a UN resolution passed by voting on 4 September 2026 (ref. ^53^).

### The lack of measured water use data

Sectoral water uses are difficult to quantify, making related data hard to find. The only way to accurately quantify real-world physical water uses is by measuring them. As an example, for the domestic sector, water use is measured by water meters or other monitoring methods^54^. Many water supply utilities in developed regions are shifting towards metering but coverage in developing regions remains low, although growing^55^. However, such data, when available, are generally not shared publicly. Water utilities often restrict water use data access because of privacy concerns, lack of standardized reporting and competitive non-disclosure agreements. Furthermore, parts of the population will not be connected to centralized water utilities. Domestic water uses in national reporting are therefore best estimates, with the quality of these assessments depending strongly on data input from water utilities and institutional capacities of countries.

Not all industrial producers meter their water use. Many abstractions are self-supplied from natural water bodies. Depending on the industrial sector of a country, the number of these connections can be very high^56^. For example, statistical surveys in the Zhejiang Province of China identified 3,282 self-supplied water industrial enterprises in their directory^56^. Large industrial water users, such as the manufacturing plants or mines, often view their water use metrics as proprietary information. Withholding these data protects them from public backlash and competitive disadvantages. When not publicly mandated, they generally do not share such data. China is an exception, where an industrial enterprise dataset includes the water use of more than 400,000 enterprises for the year 2008 (refs. ^56,57^). This database is, however, the exception and is not available for any African country nor most other countries in the world. Industrial water use amounts in national reporting are therefore generally best estimates, which do not provide information on subsectoral water use or individual companies.

For agriculture, the amount of WW connections for irrigating individual fields and providing water to livestock on individual farms throughout Africa is in millions, because of the high number of farms on the continent^58^. As farmers are generally not required to measure the water they abstract from surface or groundwater, countries have limited data to estimate the water withdrawn for agriculture, let alone in a spatially distributed way or crop- or livestock-type specific way.

National reporting on sectoral water use is therefore very limited in most countries, including Africa^59,60^. The only international database available is Aquastat of the Food and Agriculture Organization (FAO)^9^, which provides annual national sectoral WW data for agriculture (irrigation and livestock), domestic use and industry (manufacturing industry, electricity and mining). Countries are expected to provide Aquastat with national best estimates, through their statistical offices or responsible institutions, yet for Africa sectoral data are often lacking or outdated. As an example, for the statistic ‘WW for cooling of thermoelectric plants’, of 49 mainland African countries, Aquastat provides only one statistic for Zimbabwe. For the other 48 countries, no statistics are provided. Aquastat also provides only national data, and not subnational data. Its use for water management decisions is therefore very limited.

Some countries have made efforts to provide sectoral water use data with subnational spatial resolution. The United States has been at the forefront of providing such data; the last version for WWs is up to the year 2020 (ref. ^61^). However, its spatial resolution is not very high, nor is its subsectoral disaggregation. As this assessment is also restricted in measured source data, it relies heavily on modelling, a standard practice for computing sectoral water use^61^. Modelling is also essential as remote sensing cannot measure WWs, although it can help to estimate irrigation WC. Studies on sectoral water use at the national and subnational levels are scarce in the literature because of these limitations. The few existing global sectoral water use studies that cover Africa are global ones, such as refs. ^11,12^. These do not focus on Africa, however. They rely mostly on modelling and limited national statistics.

### Municipal WD

We calculated the grid cell municipal WD (WD_municipal_, equation (3)) based on different components—that is, population, a minimum domestic WD per person, the average household size, a factor accounting for other than domestic water uses within municipal water use as well as a physical water loss factor. When acquired WW data exceeded a minimum WD_municipal_, we used the former. We conducted an extensive data gathering campaign, resulting in a database with data from +450 water supply utilities, most of them through IBNET. Moreover, we searched for WW data in national sources and water supply company websites, but apart from some sources^62–73^, not much data are available. We also contacted selected water supply companies in larger cities not included in the IBNET database, but most did not want to share data because of ‘data sensitivities’. The latter is common all over the world and an important restriction when quantifying municipal WD. All water supply utility sources combined, spread over Africa, we account for more than 400 million people within their service zones, with a certain percentage not connected.

We calculated grid cell WD with following equations:

1 $$\begin{array}{c}{\mathrm{WD}}_{\mathrm{municipal}-\mathrm{noLoss}}=\mathrm{POP}\times {\mathrm{WD}}_{\mathrm{domestic}-\mathrm{noLoss}}/{\mathrm{FACTOR}}_{\mathrm{municipal}}\\ \,\mathrm{When}\,{\mathrm{WD}}_{\mathrm{municipal}-\mathrm{noLoss}}\le {\mathrm{WW}}_{\mathrm{municipal}-\mathrm{noLoss}},\,\mathrm{then}\,{\mathrm{WW}}_{\mathrm{municipal}-\mathrm{noLoss}}\\ \,\mathrm{When}\,{\mathrm{WD}}_{\mathrm{municipal}-\mathrm{noLoss}} > {\mathrm{WW}}_{\mathrm{municipal}-\mathrm{noLoss}},\,\mathrm{then}\,{\mathrm{WD}}_{\mathrm{municipal}-\mathrm{noLoss}}\end{array}$$

where WD_municipal-noLoss_ is the grid cell municipal WD without accounting for physical water loss; POP is the total population in grid cell for the year 2020; WD_domestic-noLoss_ is the minimum domestic WD per person accounting for household size; FACTOR_municipal_ is the proportion of domestic water use within municipal water use in %; WW_municipal-noLoss_ is the WW reported by water supply utility, without accounting for physical water loss:

2 $${\mathrm{WD}}_{\mathrm{domestic}-\mathrm{noLoss}}={\mathrm{WD}}_{\min }-0.5\times (\mathrm{HHsize}-1)$$

where WD_min_ is the minimum domestic WD for a one- person household, set at 50 l per person per day^74^; and HHsize is the average household size in a grid cell:

3 $${\mathrm{WD}}_{\mathrm{municipal}}={\mathrm{WD}}_{\mathrm{municipal}-\mathrm{noLoss}}/(1-\frac{{\mathrm{LOSS}}_{\mathrm{municipal}}}{100})$$

where WD_municipal_ is the grid cell municipal WD and LOSS_municipal_ is the physical water loss.

We use the Global Human Settlement Layer (GHSL)^75^ of the European Commission as the population database, which provides spatially distributed population data from 1975 to 2020 in 5-year intervals and projections to 2025 and 2030, in different spatial resolutions, from 100 m up to 30 arcsec. We use the data for the year 2020, with 30 arcsec resolution, which we aggregate to 2.5 arcmin to compute POP (equation (1)).

To compute WD_domestic-noLoss_ (equation (1)), we use equation (2), accounting for a minimum domestic WD for a one-person household (WD_min_) as well as the average household size in a grid cell (HHsize). WD_min_ is set at 50 l per person per capita per day (l cap^−1^ day^−1^). This is the value defined by the World Health Organization (WHO) as the minimum amount to meet basic health-related needs, based on water requirement for food preparation, hydration and basic hygiene^74^. This value is used in many studies^6,76^ and corresponds to ref. ^2^, which recommends a minimum value of 50 l cap^−1^ day^−1^, with 5 l cap^−1^ day^−1^ for drinking water, 20 l cap^−1^ day^−1^ for sanitation, 15 l cap^−1^ day^−1^ for bathing and 10 litres/cap/day for cooking and kitchen use. This study argues that these values may fluctuate according to climatological conditions. The WHO, for example, also indicates different values for drinking water, ranging from 3.2 l cap^−1^ day^−1^ for sedentary activity in a temperate climate to 4.6 l cap^−1^ day^−1^ for moderate physical activity at warm temperature. The latter is more appropriate as the average amount in the African context.

Household (HH) size is a notable determinant of household water use. An increase in HH size causes an increase in total household water use, whereas per capita water use decreases with an increase in HH size^77^. The decrease in per-capita use is due to many HH water uses, such as cleaning the house, washing machines, dishwashers, cooking and irrigation, being shared among the occupants of the HH. Studies in Europe, where per capita HH water use is high, have shown that these amounts can be substantial^78^. However, for low WDs such as 50 l cap^−1^ day^−1^, per capita reductions are much lower. A study in Uganda, for example, with low per capita water use, found a 0.65 l  cap^−1^ decrease for each additional HH member^74,79^. Given that average HH sizes in Africa are high compared with those of developed countries and that each member still needs water for basic services, we assume a reduction of 1 l per additional HH member, as expressed in equation (2). Subnational data for HH size (equation (2)) are provided by the Global Data Lab (globaldatalab.org), for 543 regions covering Africa, ranging from 3.08 in central Kenia to +10 in Senegal and Gambia. Calculated grid cell WD_domestic-noLoss_ amounts range from 42.5 to 49.0 l cap^−1^ day^−1^.

FACTOR_municipal_ (equation (1)) accounts for the fact that a certain proportion of municipal water is for non-domestic use, such as water for small businesses, services such as schools or hospitals, and others. Generally, this proportion is about 20–30% of municipal WD, as confirmed by most African water utilities in IBNET. Here, we used IBNET data, which were extrapolated to regions in which data are missing. FACTOR_municipal_ is thus about 70–80%.

LOSS_municipal_ (equation (3)) is the physical water loss in municipal water use, defined as the percentage of water physically lost between what a water supply utility abstracts from the natural system and what it provides to end users. This physical loss is the result of leakage due to old pipes or poor maintenance, but is also caused by pipe breaks. IBNET does not provide many statistics on physical loss in African utilities, but it provides statistics on non-revenue water (NRW) for many utilities. Apart from physical loss, NRW, for example, includes unbilled water or water theft. For utilities in which LOSS_municipal_ were not available, we use utility NRW statistics for our calculations. We assume that 75% of NRW is actual LOSS_municipal_, an amount that can be justified by different sources^59^. Representative utility data are extrapolated to regions for which data are missing.

Where no utility WW data were available, we used national data from Aquastat^9^ and/or national reports. The national municipal WW data Aquastat provides are production data (hence similar to WD_municipal_ and not WD_municipal-noLoss_ in equation (3)); this is because Aquastat reports WW measured at the point of withdrawal, not at the point of delivery. Aquastat also theoretically includes only statistics on WW by utilities, so WW from piped connections, although some countries might in praxis add amounts from non-piped coverage. Although Aquastat provides values for almost all African countries for 2020, most of these values are ‘imputed’ values, meaning they originate from a data entry for an earlier year, with many dating back to 2005 or even 2000. We calculate national WW for the most recent year for which year Aquastat provides a data entry or based on a national source as follows:

4 $${\mathrm{WW}}_{\mathrm{connected},\mathrm{yr}}=\frac{{\mathrm{WW}}_{\mathrm{national},\mathrm{yr}}\,}{({\mathrm{POP}}_{\mathrm{national},\mathrm{yr}}\times 365)}\times {\mathrm{POP}}_{\mathrm{connected},\mathrm{yr}}\times (1-\frac{{\mathrm{LOSS}}_{\mathrm{municipal}}}{100})$$

where WW_connected,__yr_ is the WW of population connected for a certain year in l cap^−1^ day^−1^; WW_national,__yr_ is the national WW reported by Aquastat or national source in m^3^ for a certain year; POP_national,__yr_ is the national population for a certain year; POP_connected,__yr_ is the percentage of national population with pipe connection for a certain year, data provided by the WHO/UNICEF Joint Monitoring Programme on household drinking water, sanitation and hygiene^3^.

As an example, the most recent value for Botswana in Aquastat for WW_national,__yr_ is 109 Mio m^3^ yr^−1^ for the year 2020 (ref. ^11^), with 91% of the population connected to piped supply^3^. Equation (4) becomes (109 Mio × 1,000 litres/(2,546,402 × 365 days)) × 0.91 × (1 − (28/100)) = 93 l cap^−1^ day^−1^. IBNET provides a value of WW_municipal-noLoss_ for Gaborone of 89 l cap^−1^ day^−1^. This amount is very similar to the calculated national amount, so that the Aquastat value can be regarded as reliable. However, Aquastat data that are imputed from 2005 or earlier, have proven to be unreliable in many cases. As an example, for Burkina Faso an imputed WW_national,__yr_ amount from 2005 of 376 Mio m^3^ yr^−1^ (ref. ^11^) is provided, with 22% of the population connected to piped supply for the same year^3^. Equation (4) becomes (376 Mio × 1,000 litres/(13,876,127 × 365 days)) × 0.22 × (1 − (28/100))  = 232 l cap^−1^ day^−1^. IBNET provides a value of WW_municipal-noLoss_ for Ouagadougou of 41 l cap^−1^ day^−1^. This confirms that the Aquastat amount is unreliable, so it is disregarded in this analysis. Instead, the IBNET amount for Ouagadougou is assumed for the national population with piped access, which is 30% in 2020. A full list of calculated national WW_connected,__yr_ amounts is provided in Supplementary Table 2.

These national WW_connected,__yr_ amounts are then spatially distributed over the population within a country according to the high-resolution spatial dataset on piped-access (year 2017) provided in ref. ^17^, except for the areas in which IBNET WW data (WD_municipal-noLoss_) are available. When WD_municipal-noLoss_ > WW_municipal-noLoss_ or grid-level WW_connected,__yr,_ WD_municipal-noLoss_ is used; otherwise, WW_municipal-noLoss_ or grid-level WW_connected,__yr_ is used. Then, physical water loss is added as shown in equation (3).

For population not connected to piped water access, the national percentage of population with piped access is spatially distributed to the grid cell level by means of the same dataset as in ref. ^17^. For this part of the population, WD_municipal_ (equation (3)) is applied.

People not connected to piped water access may have other kinds of access, such as yard taps, public taps, wells, boreholes or surface water. Often these sources are of poor quality and may require substantial round-trip travel time for water collection, especially in many African regions. Generally, people are not able to collect large quantities of water from these sources, not reaching the minimum amount of 50 litres per person per day^74^ we set in our analysis. They are thereby deprived of a minimum drinking WD and are defined as a population without piped access.

### Irrigation WD

Irrigation WD was calculated by means of modelling WC for 46 different SPAM crops and main crop groupings for the years 2020 and 2010 (ref. ^13^). SPAM2020 was released by the International Food Policy Research Institute in 2024. The SPAM database provides crop harvested area and production data at a spatial resolution of 5 arcmin. WC in these calculations is defined as consumptive WD; that is, the required WC amounts are met for the crop. For 2010, we used the SPAM2010 database. SPAM has released global data, thus covering the whole of Africa, for the years 2020, 2010, 2005 and 2000, not for years in between.

We used the global gridded model CropGBWater, an open-access Pythton script which runs with a daily time step^80^. We ran the model with climate data from 2018 to 2022, representing the year 2020 as an average, and for 2008 to 2012, representing the year 2010 as an average. We used this multi-year average approach to level out climatological fluctuations, similar to that in ref. ^81^. We used only open-access input data, such as reference evapotranspiration (Eto) data from the AgERA5 dataset of FAO^82^, precipitation from ref. ^83^, total available water capacity from ref. ^84^, curve number from ref. ^85^ and crop coefficient *K*_c_ as well as length of growth stages from ref. ^86^. Crop calendars are from reliable sources, specifically the SAGE^87,88^ and MIRCA2000^89^ datasets, as well as some additional regional sources^90,91^.

The 46 SPAM crops for which we model WC are as follows: wheat, rice, maize, barley, small millet, pearl millet, sorghum, other cereals, potato, sweet potato, yams, cassava, other roots, bean, chickpea, cowpea, pigeon pea, lentil, other pulses, soybean, groundnut, coconut, oil palm, sunflower, rapeseed, sesame seed, other oil crops, sugarcane, sugar beet, cotton, other fibre crops, Arabic coffee, robust coffee, cocoa, tea, tobacco, banana, plantain, citrus, other tropical fruit, temperate fruit, tomato, other vegetables, rubber, rest of crops.

Modelled daily blue WC results per crop were aggregated to annual amounts (WC_crop-typeX_, equation (1)). By accounting for irrigation efficiency (IE), grid cell WD amounts per crop are computed as follows:

5 $${\mathrm{WD}}_{\mathrm{crop}-\mathrm{typeX}}={\mathrm{WC}}_{\mathrm{crop}-\mathrm{typeX}}\times 100/\mathrm{IE}$$

where WD_crop-typeX_ is the WD of crop type X in a grid cell; WC_crop-typeX_ is the WC of crop type X in a grid cell.

IE measures the ratio between irrigation WC and WD. According to an FAO publication of 2012 (ref. ^92^), as of 2010, the mean IE of the world was approximately 56%. For our Africa study, we provide the best estimates of IE based on several sources^92–94^, including regional and local studies in Algeria^95–98^, Botswana^99^, Burkina Faso^100^, Côte d’Ivoire^101^, Egypt^102–104^, Ethiopia^104–108^, Ghana^109^, Kenya^104^, Libya^110,111^, Malawi^112,113^, Mali^114^, Morocco^115^, Niger^114^, South Africa^116^, Sudan^104^, Tunisia^117^ and Uganda^104^.

### Livestock WD

Livestock WC was calculated by means of the global database on livestock distribution (Gridded Livestock of the World or GLW v.4, resolution 5 arcmin) for the years 2020 and 2010 (refs. ^48,49^). We account for a total of six livestock types (buffaloes, cattle, chickens, goats, pigs and sheep). For all livestock types, the data were recently (in 2024) updated for the year 2020. Grid cell head counts were multiplied by unit water intensities for drinking and service water, resulting in grid cell WD amounts per livestock type (equation (6)):

6 $${\mathrm{WD}}_{\mathrm{livestock}-\mathrm{typeX}}={\mathrm{HEAD}}_{\mathrm{livestock}-\mathrm{typeX}}\times ({\mathrm{WI-D}}_{\mathrm{livestock}-\mathrm{typeX}}+{\mathrm{WI-S}}_{\mathrm{livestock}-\mathrm{typeX}})$$

where WD_livestock-typeX_ is the WD of livestock type X in a grid cell; HEAD_livestock-typeX_ is the head count of livestock type X in a grid cell in the year 2020; WI-D_livestock-typeX_ is the drinking water intensity for livestock type X; WI-S_livestock-typeX_ is the service water intensity for livestock type X.

Unit water intensities (drinking and service water) per livestock type were obtained from different sources^118,119^. In terms of head count, for the year 2020, chickens show the highest numbers in Africa (2,167 million), followed by goats (490 million), sheep (414 million), cattle (372 million) and pigs (44 million). For the year 2010, the numbers are lower for chickens (1,669 million), goats (329 million), sheep (309 million), cattle (285 million) and pigs (31 million).

### Electricity

We used the spatially most detailed database on power plant WD in Africa, developed in ref. ^40^. This database incorporates WD of 2,534 individual power plants for the year 2020, including 1,447 fossil (coal, oil and natural gas), 1,071 renewable (wind, sun, biomass, geothermal and hydropower with the distinction between reservoir and run-of-river hydropower) and 16 other (waste heat and nuclear) power plants. Power plants are categorized according to applied fuel, operation cycle, infrastructure, cooling system and local climate. This open access database is the result of an important data mining effort using only open access data. As we include mainland Africa and Madagascar but not small African islands, the total number of power plants is reduced to 2,357.

We identified the year of first operation for each power plant through multiple sources such as GEM WIKI, to know which power plants were operational by the year 2010. As such, we can calculate the WD for electricity in 2010. We find that after 2010, 846 additional power plants became operational until 2020. In 2010, 1,511 power plants were operational.

We also account for the WD of hydropower, because of its large current and growing water use in Africa, which ref. ^40^ quantified at 23,038 Mm^3^ in 2020. However, hydropower is an in-stream water user, whereas all other WD sectors and subsectors are off-stream water users. The WD is calculated based on the evaporation of open water surfaces (for example, reservoirs)^40^. This is in line with Aquastat^9^, which also reports evaporation of reservoirs as a separate amount.

This evaporation does affect EFs, in the sense that the reservoir water is evaporated and therefore not available downstream, potentially contributing to the violation of downstream EFs and resulting in diminished ecosystem services. Our assessment does not account for the additional negative effect of altered flow regimes and dam barriers, which block fish migration when fish passage is not ensured, leading to fragmented habitats, reduced populations and interrupted spawning cycles^120,121^.

### Mining

To quantify the WD of mines in Africa, three steps are included. In the first step, we identified operational mines (only legal mines, not illegal mines) with specifics such as geolocation, main commodity and production data for this commodity for the years 2020 and 2010, resulting in 682 individual mines with reliable data. In a second step, we identified the water intensity of the commodities for all mines, based upon literature values and national reports. In a third step, we calculated the WD for each mine (equation (7)):

7 $${\mathrm{WD}}_{\mathrm{mine},\mathrm{comX}}={\mathrm{PROD}}_{\mathrm{mine},\mathrm{comX}}\times {\mathrm{WI}}_{\mathrm{comX}}$$

where WD_mine,__comX_ is the WD of a certain mine for commodity X. We included 34 commodities in total; PROD_mine,__comX_ is the production of commodity X in the year 2020 and 2010 for a certain mine; and WI_comX_ is the water intensity of commodity X.

In the first step, we analysed existing spatially distributed mine geodatasets, such as the ‘Africa Power–Mining Database’^122^ of the World Bank or the geospatial mining datasets of the United States Geological Survey^123,124^, to identify operational mines.

We selected 34 main commodities: aluminium, bauxite, black coal, chromium, coal, cobalt, copper, diamond, fluorspar, gold, ilmenite, iron ore, lead, lithium, manganese, nickel, nickel from N-C sulfide, palladium, PGM, phosphate, phosphate rock, platinum, potash, rare elements, rhodium, rutile, silver, titanium dioxide, tungsten, uranium, vanadium, zinc, zircon and zirconium.

For each mine and commodity, we gathered production data representative of the years 2020 and 2010. Production data were primarily sourced from annual and integrated reports, fact sheets from countries or mining companies, national government databases, company websites, and the United States Geological Survey database^123^. The production reporting of each commodity as ore, concentrate or metal was taken into account.

The COVID-19 pandemic markedly affected mining outputs in 2020, with many mines reporting reduced production levels. To address this issue, we often used data from the nearest available year (2018, 2019, 2021, and so on) when 2020 values were considered too low. Apart from individual mining data, national production data for each country were collected commodity-wise. To estimate production for mines with missing data, the total reported production for a commodity from all mines in a country was deducted from the national production value of that country, and the resulting value was evenly distributed across the mines with missing data. An overview of all sources for production is provided in Supplementary Data 5.

In the second step, we collected water intensities for all 35 commodities, accounting for the fact whether production is reported as ore, concentrate or metal, from the scientific literature and reports^60,125–132^. An overview of water factors for each commodity is provided in Supplementary Data 5.

### Manufacturing industry

Manufacturing industry WD is the total volume of water used by the manufacturing industry if sufficient water is available. It is calculated by different authors^12^ by accounting for GVA for the manufacturing industry and its water intensity (WI = WD per unit GVA).

Manufacturing WD depends on the volume and nature of industrial activities. The manufacturing sector is rather diverse, as it includes different subsectors (for example, paper and pulp, chemical industry, machinery, food and beverages industry) with varying degrees of WI. It is therefore important to account for the WI of a certain subsector and its importance within the national economy. According to ref. ^12^, subsectoral manufacturing output GVA is not available from statistics, so these authors used national accounts of the whole manufacturing industry sector. We, however, found and used an international GVA database on subsectors of the manufacturing industry, making our assessment more detailed compared with ref. ^12^. This database is the INDSTAT, ISIC database (revisions 3 and 4), published by the United Nations Industrial Development Organization (UNIDO)^18^. It provides national GVA amounts, according to the International Standard Industrial Classification of All Economic Activities (ISIC). Within ISIC, the manufacturing industry has code D (Total manufacturing), with ISIC divisions 10–33. We used the average annual GVA_ind,__subsectorX_ amount for the 3-year period 2019–2021 as representative for 2020, to balance the influence of the COVID pandemic. An overview of these amounts is provided in Supplementary Data 6.

The WD of the manufacturing industry is the sum of the WD of all subsectors:

8 $${\mathrm{WD}}_{\mathrm{ind},\mathrm{subsecto}\mathrm{rX}}={\mathrm{GVA}}_{\mathrm{ind},\mathrm{subsector}{\rm{X}}}\times {\mathrm{WI}}_{\mathrm{ind},\mathrm{subsector}{\rm{X}},{\rm{A}}}$$

where WD_ind,__subsectorX_ is the WD of a certain subsector X of the manufacturing industry. We included or aggregated to 8 subsectors (4 individual and 4 aggregated subsectors); GVA_ind,__subsectorX_ is the national GVA of a certain subsector X of the manufacturing industry for the year 2020 (ref. ^18^); WI_ind,__subsectorX,__A_ is the WI of a certain subsector X of the manufacturing industry, accounting for the development stage A of the country in the years 2020 and 2010. The development stage is defined as the four categories of income classifications of the World Bank based on their gross national income (GNI) per capita, that is, low income (GNI ≤ 1,045), lower-middle income (GNI = 1,046–4,095), upper-middle income (GNI = 4,046–12,695), high income (GNI > 12,695). The latter is included to account for the technical level of a country, as water intensities decrease with higher income (and resulting technical level)^12^.

We account for or aggregate to the following subsectors: aggregated subsector ‘Food and beverages’, comprising ‘Food products’ (ISIC Rev4 code 10) and ‘Beverages’ (ISIC Rev4 code 11); aggregated subsectors ‘Clothing’, comprising ‘Textiles, Wearing apparel, Leather and related products’ (ISIC Rev4 codes 13, 14, 15, respectively); subsector ‘Paper and paper products’ (ISIC Rev4 code 17); subsector ‘Coke and refined petroleum products’ (ISIC Rev4 code 19); aggregated subsector ‘Chemicals and pharmaceuticals’, comprising ‘Chemicals and chemical products; Pharmaceuticals, medicinal chemicals, etc.’ (ISIC Rev4 codes 20 and 21, respectively); subsector ‘Basic metals’ (ISIC Rev4 code 24); subsector ‘Motor vehicles, trailers and semi-trailers’ (ISIC Rev4 code 29); aggregated subsector ‘other’, comprising ‘Tobacco products; Wood products, excluding furniture; Printing and reproduction of recorded media; Rubber and plastics products; Other non-metallic mineral products; Fabricated metal products, except machinery; Computer, electronic and optical products; Electrical equipment; Machinery and equipment n.e.c.; Other transport equipment; Furniture; Other manufacturing’ (ISIC Rev4 codes 12, 16, 18, 22, 23, 25, 26, 27, 28, 30, 31 and 32, respectively).

We defined WI_ind,__subsectorX,__A_ values based on subsector WI values from the literature. EUROSTAT provides a comprehensive subsector WI database for countries of the pan-European area, including for countries such as Bulgaria and Turkey^133^. Subsector WI amounts for latter countries for their developmental stage in earlier years can be used for African countries that have the same development stage in the year 2020. With development, unit WI decreases, as shown by EUROSTAT data^133^ but also for countries such as the United States or China^134^. In the United States, WI_ind_ decreased from 217 m^3^/USD1,000 in 1970 to 14 m^3^/USD1,000 in 2000 to 9 m^3^/USD1,000 in 2015 (ref. ^135^). In Germany, WI_ind_ in 2019 was as low as 6 m^3^/USD1,000, whereas in Turkey, it was 31 m^3^/USD1,000 and in Bulgaria 23 m^3^/USD1,000 in 2020.

Other data sources we used include Statistics Canada^136^ and a report on industrial water use of the textile and leather industries in Bangladesh, the main global clothing producer, which shows a WI_ind_ value for the clothing industry of 151 m^3^/USD1,000 (ref. ^137^). This high value confirms the high water intensity of the textile industry and can be regarded as relevant for many countries in the Global South. The food and beverages industry of Uganda shows a WI_ind_ of 23 m^3^/USD1,000, as listed in its system of environmental economic accounting 2015–2018 (ref. ^138^). For Egypt, the value is 15 m^3^/USD1,000, very similar to that of Turkey (17 m^3^/USD1,000 in 2020)^133^, Canada (13 m^3^/USD1,000 in 2011)^136^ or Australia (14 m^3^/USD1000 in 2020)^139^. An overview of WI_ind,__subsectorX,__A_ values is provided in Supplementary Data 6, showing different levels of WI_ind,__subsectorX,__A_.

In line with ref. ^140^, industrial WDs were downscaled from the national to grid cell values using the NASA Black Marble night-time light intensity map^141^, which is preferable compared with downscaling according to population density. We used product VNP46A, a dataset at 15 arcsec resolution (500 m at the equator), and rescaled it to the same resolution of municipal WD (2.5 arcmin). For 2020, we used the annual VNP46A of the year 2020. For 2010, we used the annual VNP46A of the year 2012, because earlier data of the NASA Black Marble night-time light intensity map^141^ are not provided. National industrial WDs were allocated based on the relative light intensity per grid cell for each country. The resulting raster is provided as Supplementary Data 7.

We acknowledge that night-time light intensity maps can overlook rural informal sectors, as they primarily capture artificial lighting associated with electrification, which is less prevalent in rural, low-income areas^142^. Rural informal sectors, such as home-based manufacturing, often operate without much electric lighting, rendering them invisible to satellite sensors. Outside urban centres, most night-time lighting tends to serve household needs—cooking, studying and socializing—rather than the manufacturing or commercial production. This limitation is inherent to all studies. Nevertheless, according to ref. ^143^, night-time light should not be underestimated as a method to overcome the lack of population census data and socioeconomic surveys at very fine levels and outside cities in middle- and low-income countries. Our assessment is to be regarded as best estimate.

### WD related to water resources

We quantified the relation of total WD to renewable water availability in the river basins of Africa. We defined renewable water availability as natural renewable water minus EFs:

9 $$\text{renewable water availability}\,=\,\text{natural renewable water}-{\rm{EF}}$$

Natural renewable water at a spatial resolution of 0.5° (which equals 55.5 km at the equator) was taken from two global models: WaterGAP2-2e^144^ and LPJmL5-7-10-fire^145,146^. We took two models to account for uncertainty in the output of global hydrological models. Their output is provided in ISIMIP (Inter-Sectoral Impact Model Intercomparison Project), simulation round ISIMIP3a, under the ‘nowatermgt’ run, which models naturalized flow (without human water abstraction or reservoirs). We used monthly outputs to be able to calculate EFs, for the period 2010 to 2020, a common practice to account for interannual variation.

We use two EF methods: one accounting for high EF protection, that is, the presumptive standard in ref. ^15^. It defines 80% of the natural flow as EF. The remaining 20% is considered as water available for human use. This presumptive standard is supported by empirical studies showing that flow alterations within 20% support native fish species and flow alteration beyond this level strongly affects biodiversity and ecosystem structure and function^147^. It is widely used in many water management studies^5,76,148^. The other EF method is the VMF method^16^, which accounts for lower EF protection. During low-flow months (when long-term mean monthly streamflow (MMF) is ≤40% of the long-term mean annual flow (MAF)), 60% of MMF is allocated to EFs; in high-flow months (MMF > 80% of MAF) the EF share is 30% of MMF; otherwise it is 45%. This method was tested by means of case studies of locally assessed EFs and is also widely used^5^. By using these two EF methods, we provide an upper and lower range in renewable water availability (equation (9)), because one represents high and the other lower EF protection.

We aggregated monthly amounts to the annual level for the African river basins. We then related total WD to annual renewable water availability to obtain a level of water stress. This approach is in line with SDG indicator 6.4.2 (ref. ^149^), and its statistics are provided by Aquastat. Values exceeding 100% theoretically mean that EFs are violated. More sophisticated water stress assessments also look at consumptive WD, on the monthly level and/or the sub-basin level, as discussed in ref. ^10^ and analysed by several studies^5,150,151^. However, using WD on the annual level provides a first indication of water stress and is in line with Aquastat and general SDG reporting.

We do not account for regional or local EF assessments, as they do not cover the entire continent of Africa and because the two methods we use are well established to provide an upper and lower range in renewable water availability. However, these regional EF methods exist, especially in southern Africa, where much effort has been done to identify these methodologies^152–154^.

The natural renewable water assessments we use, that is, WaterGAP2-2e^144^ and LPJmL5-7-10-fire^145,146^, include renewable groundwater. They do not account for fossil groundwater^10^, primarily found in vast, non-renewable aquifers beneath the Sahara. Groundwater is an important buffer for the availability of freshwater throughout the year.

### Validation

Few studies provide spatially distributed sectoral WD data covering the whole of Africa. We validate our results with data from Aquastat^9^, through comparison with other studies and through additional modelling approaches.

Other studies include the study in ref. ^12^, which quantified municipal and industrial WDs for the year 2010 on a global level; ref. ^11^, which quantified sectoral WDs for the year 2010 on a global level in a spatial resolution of 0.5° (50–55 km); ISIMIP^155,156^, which quantified sectoral WDs for the years 2010 and 2020 on a global level in a spatial resolution of 0.5°; and ref. ^157^, which quantified industrial WDs for the years 2005, 2010 and 2015 on a global level in a spatial resolution of 0.5°. For specific sectors, we use selected high-quality studies^118,158,159^.

A previous study^12^ computed the WD for only three sectors (municipal, manufacturing industry and electricity) at a resolution of 5 arcmin until 2010, without accounting for subsectors, yet did not provide their data in open access. This study computed multi-sectoral water use up to 2010, in much coarser resolution (0.5°, that is, about 55 km from the equator) and by simple downscaling of the sectors mining^11^, manufacturing industry^11,12^ and electricity^11^ based on population density. The study did not account for subsectors, individual crops or livestock types. As first-time novelties, we explicitly identify and locate mines and individual electricity power plants; we account for actual water supply data of more than 450 African water supply utilities; we assess the manufacturing industry at the subsectoral level; and we provide an assessment for all sectors for the recent year 2020 as well as 2010 at the highest spatial resolution.

For additional validation of our manufacturing industry WD results, we conducted industrial WD quantifications for selected industrial subsectors where outputs are available in weight quantities.

10 $${\mathrm{WD}}_{\mathrm{ind},\mathrm{subsecto}\mathrm{rX}}={\mathrm{QUANTITY}}_{\mathrm{ind},\mathrm{subsectorX}}\times {\mathrm{WI}}_{\mathrm{ind},\mathrm{subsectorX},{\rm{A}}}$$

where

- WD_ind,__subsectorX_ is the WD of a certain subsector X of the manufacturing industry;
- QUANTITY_ind,__subsectorX_ is the national weight quantity of a certain subsector X of the manufacturing industry for the year 2020; and
- WI_ind,__subsectorX,__A_ is the WI of a certain subsector X of the manufacturing industry, expressed in m^3^ per tonne or litres per kg.

The food and beverage processing industry is a subsector in which these data are available at the national level, through the FAO Food Balance Sheets (FAO-FBSs)^34^. An FAO-FBS provides a comprehensive picture of the agrifood situation of a country in a specified reference period, showing the pattern of the food supply and utilizations of a country. Food and beverages processed in the national food and beverages processing industry are provided under the element ‘processed’. We used these quantities as QUANTITY_ind,__subsectorX_. FAO-FBSs does not provide values for all African countries. The specific subsector WI for food and beverage processing is set at an average of 8 m^3^ per tonne, a value supported by several literature sources, including African ones^160–162^. For the subsectors, ‘clothing’, industry output statistics are generally provided only in monetary output. We found only one reliable weight statistic for Morocco, a large textile and clothing producer on the African continent—that is, an annual production of 950,000 tonnes (ref. ^163^). The textile and clothing industry uses an average of 100–300 m^3^ of water per tonne of finished fabric (average 200 m^3^ of water per tonne), which is a wide range depending on technique and material used^164–167^.

For the subsector ‘paper and paper products’, we found a reliable production statistic for South Africa, that is, 1,891,283 tonnes for 2020 (ref. ^24^). South African and international data show a rather wide range in the water intensity of the paper processing industry, with an average of about 70 m^3^ per tonne, depending on the kind and state of processing mills and the product produced^168,169^. Modern mills have lower water intensities, and older mills have higher values.

The detailed discussion is presented in the Supplementary Information section 3.

### Uncertainty and sensitivity

Similar to any modelling study, different assumptions have been made, which lead to uncertainty in the WD amounts we present. We therefore conduct selected uncertainty and sensitivity analyses.

We account for uncertainty in water availability by using two different EF methods, as described in the previous section “WD related to water resources”. The resulting variation in WDs related to available renewable water resources in African river basins is presented in Fig. 6.

To compute crop-specific irrigation WDs, we used the factor IE in each grid cell (equation (5)). Although we provide IE best estimates based on several sources, including local studies, IE has a certain level of uncertainty. To account for this, we conducted a spatial Monte Carlo analysis by randomly varying IE (the random variable) amounts between ±20% and their effect on irrigation WDs for the year 2020, while accounting for the spatial distribution of individual irrigated areas and Tobler’s first law of geography^170^. To do the latter, we created clusters of points that are both adjacent (eight-way connectivity) and have similar nominal IE values. During the Monte Carlo simulation, a single random IE value is assigned to all points within each cluster (an irrigation area) in every iteration (1 million in total), preserving spatial autocorrelation in the generated uncertainty. For the range of IE uncertainty (±20% of the nominal value), we use a uniform distribution. A uniform distribution was used because all values within the ±20% range are considered equally likely. The resulting irrigation WD curve is presented in Supplementary Information section 4.

We also conduct a sensitivity analysis for municipal WDs in the year 2020, by varying the factors LOSS_municipal_ (physical water loss) and FACTOR_municipal_ (the proportion of domestic water use within municipal water use in %), as used in equations (1)–(4). We vary LOSS_municipal_ as well as 100 − FACTOR_municipal_ by −20% and +20% for all 1.4 million grid cells as a sensitivity analysis. We choose 100 – FACTOR_municipal_, because other domestic water uses are in the range 20–30% on average. It is realistic to vary these amounts by −20% and +20%. Varying the domestic part—on average in the range 70–80%—by −20% and +20%, would be a much wider variation (for example, adding 20% to 80% results in 96%, leaving only 4% for other uses, which can be perceived as extreme and rare). Resulting municipal WD amounts (piped, non-piped and total) are presented in Supplementary Information section 4.

To account for uncertainty in individual mine production in the year 2020 during the COVID-19 pandemic, we conduct a sensitivity analysis based on individual mine production amounts for the years 2019, 2020 and 2021. The results are presented in Supplementary Information section 4.

## Online content

Any methods, additional references, Nature Portfolio reporting summaries, source data, extended data, supplementary information, acknowledgements, peer review information; details of author contributions and competing interests; and statements of data and code availability are available at https://doi.org/10.1038/s41586-026-11199-8.

## Supplementary information

Supplementary Information This file contains Supplementary Information sections 1–4 (General, Comparison WD years 2020 and 2010, Validation and Uncertainty and sensitivity), including Supplementary Tables 1–13 and Supplementary Figs. 1–6. Supplementary Datasets 1–11 are hosted separately on Zenodo^171^ (https://doi.org/10.5281/zenodo.15461755). A detailed description of these supplementary datasets from this study can be found at Zenodo.

## Figures and Tables

**Extended Data Table 1:**
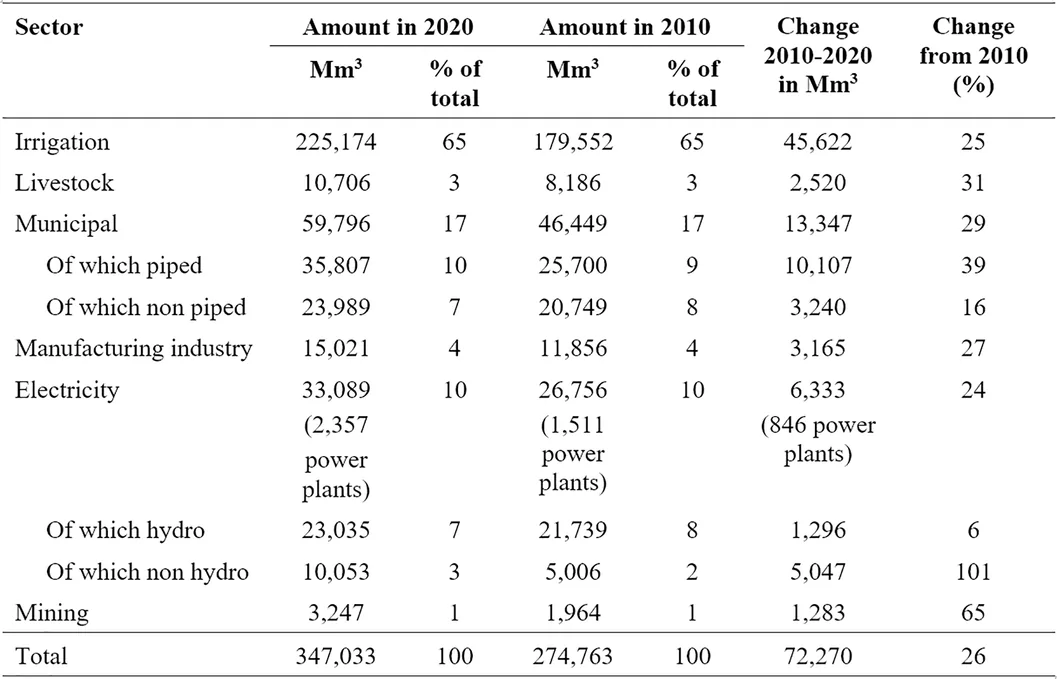
Comparison sectoral WD amounts 2020 and 2010

**Extended Data Table 2:**
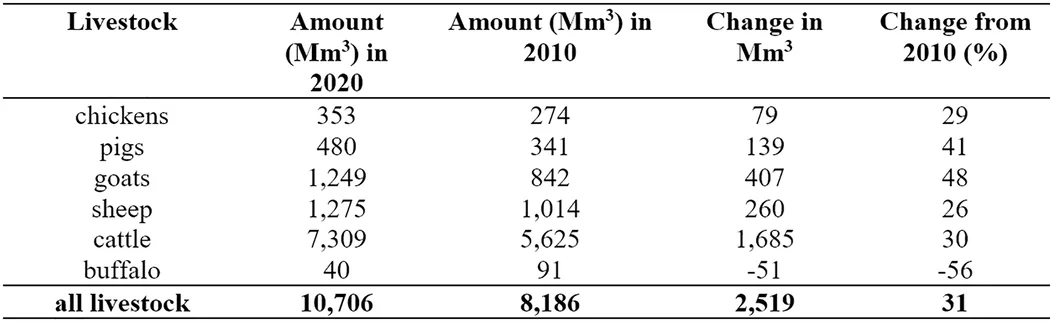
Comparison livestock specific WD amounts 2020 and 2010

**Extended Data Table 3:**
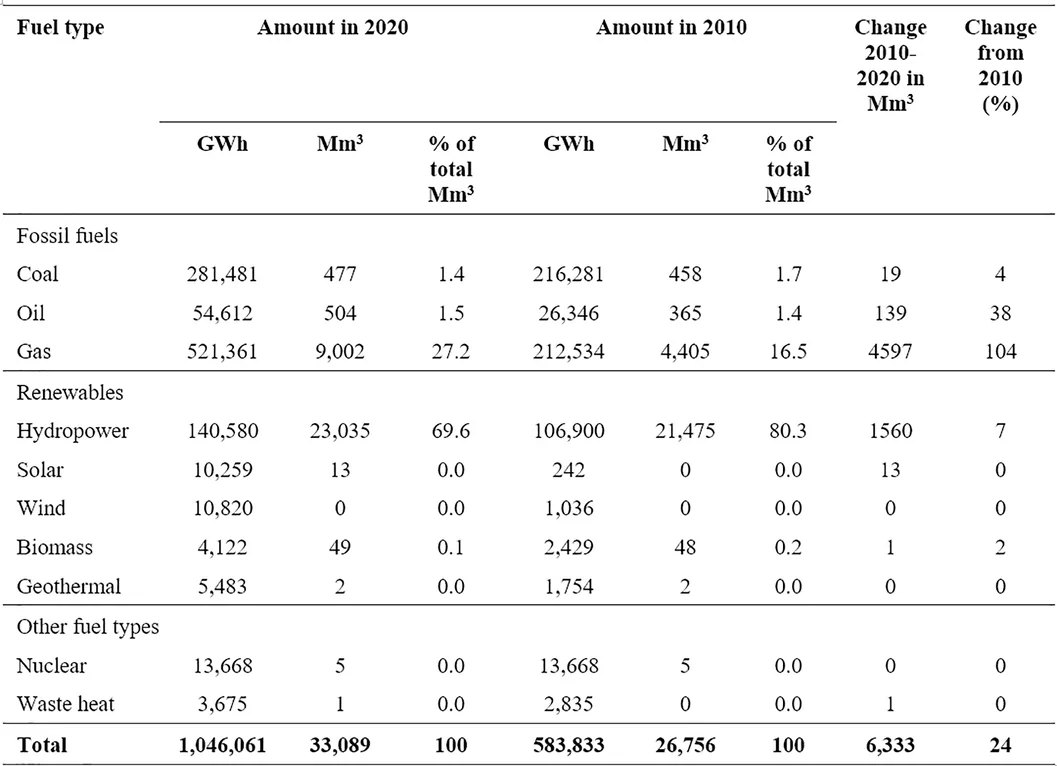
Comparison electricity WD amounts per fuel type 2020 and 2010

**Extended Data Table 4:**
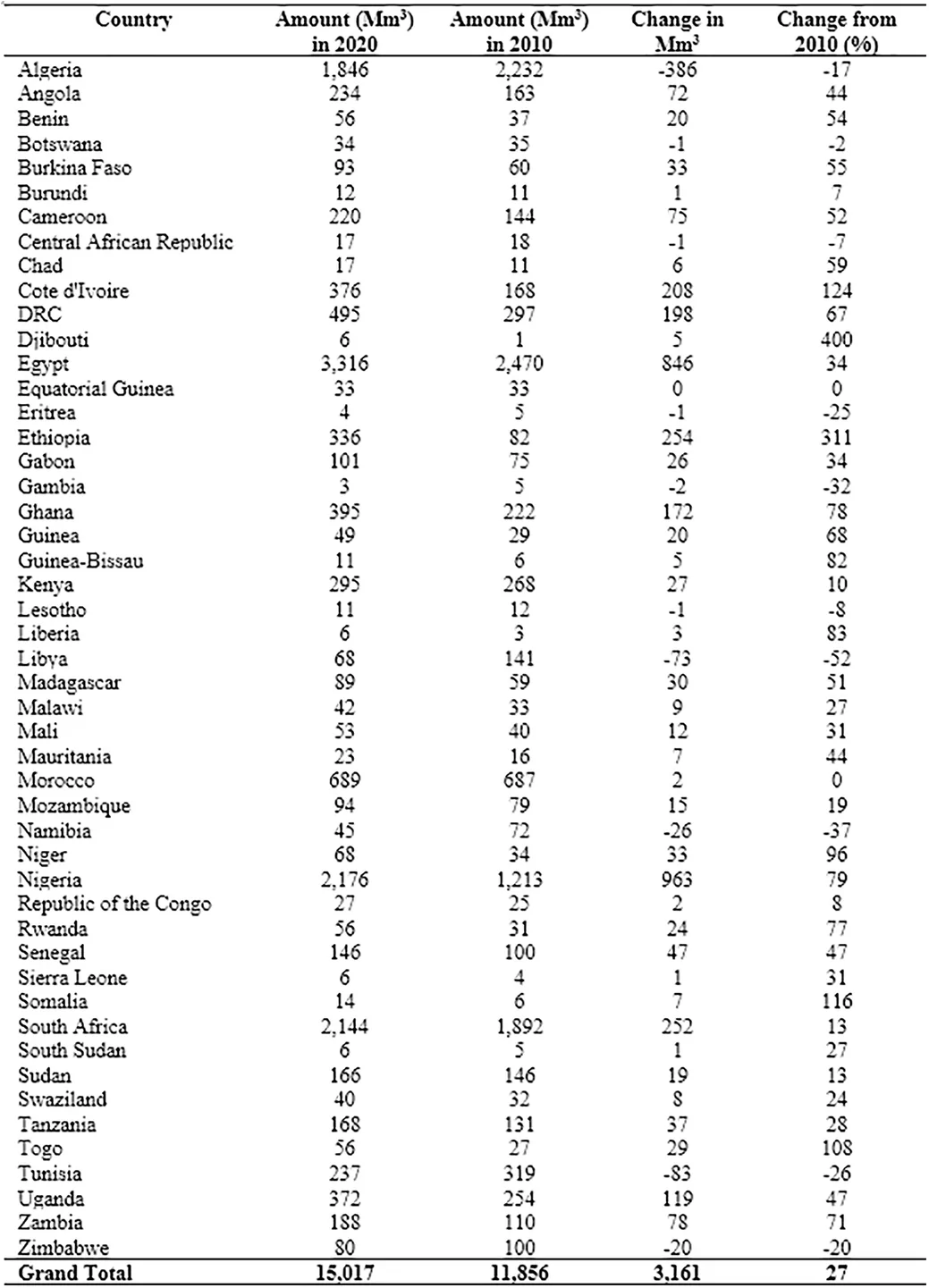
Comparison national manufacturing industry WD amounts 2020 and 2010

## Data Availability

We used only open access input data. All supplementary datasets 1–11 are available at Zenodo (https://doi.org/10.5281/zenodo.15461755) (ref. ^171^). A detailed description of these supplementary datasets from this study is available at Zenodo.

## Code availability

We did not use or develop code in this assessment
