# Supplementary material for: Spatial and sectoral water demand assessment for the African continent
Source: Nature. 2026 Oct 28;659(8140):386–94. doi: 10.1038/s41586-026-11199-8 (PMC13754605; doi:10.1038/s41586-026-11199-8)
Supplement: Supplementary file 1 [file 41586_2026_11199_MOESM1_ESM.pdf]

nature portfolio

https://doi.org/10.1038/s41586-026-11199-8

Supplementary information

Spatial and sectoral water demand assessment for the African continent

In the format provided by the authors and unedited

Spatial and sectoral water demand assessment for the African continent

Supplementary Information

Davy Vanham[1], Dahami Gunathilake[1], Bhawani Gunasekara[1], Seifu Tilahun[2,3], Youssef Brouziyne[4], Fasikaw A. Zimale[3], Monika Rawat[5], Raphael Vanham[6], Mohamed El Hafyani[7,8], Asma Thowfeek[1], Sithara Kumarasinghe[1], S.D. Vaca-Jiménez[9,10], P.W. Gerbens-Leenes[10], Simon Langan[1]

[1] International Water Management Institute (IWMI), Colombo, Sri Lanka

[2] International Water Management Institute (IWMI), Accra, Ghana

[3] Bahir Dar University, P.O. Box 26, Bahi Dar, Ethiopia

[4] International Water Management Institute (IWMI), Cairo, Egypt

[5] University of Southern Queensland, Queensland- 4350, Australia

[6] Department of Mathematics, University of Innsbruck, Innsbruck, Austria

7 National Thematic Institute for Scientific Research – Water, Ibnou Zohr University, Agadir, 80000, Morocco

[8] Applied Geology and Geo-Environment Laboratory, Faculty of Sciences, Ibnou Zohr University, Agadir, 80035, Morocco

[9] Departamento de Ingeniería Mecánica, Escuela Politécnica Nacional, Ladrón de Guevara E11·253, 01-17-2759, Quito, Ecuador

[10] Integrated Research on Energy, Environment and Society (IREES), University of Groningen, Groningen, The Netherlands

The Supplementary Information consists of different sections:

- Section 1: General
- Section 2: Comparison WD years 2020 and 2010
- Section 3: Validation
- Section 4: Uncertainty and sensitivity

1. **General**

Supplementary Table 1: Overview main data sources used in this study

**Data**	**Source**
Aquastat national statistics on sectoral water withdrawal	FAO's Aquastat[9]
Gridded population data years 2020 and 2010, resolution 30 arcsec (about 1 km), which we aggregate to 2,5 arcmin (about 5 km)	European Commission's Global Human Settlement Layer (GHSL)[76]
IBNET data from +450 water supply utilities. Data on water withdrawal, connected population, water loss, proportions domestic and non-domestic water withdrawal, …	IBNET (the International Benchmarking Network)[11]<br>Many additional sources as listed in Supplementary Table 2
Subnational data on household size, for 543 regions covering Africa	Global Data Lab[81]
High-resolution spatial dataset on piped-access (year 2017)	Deshpande et al[18,82]
National population with piped connection for a certain year	WHO/UNICEF Joint Monitoring Programme (JMP) on household drinking water, sanitation and hygiene[3]
SPAM crops and major crop groupings for the years 2020 (46 crops) and 2010 (42 crops), spatial resolution of 5 arc minutes (about 10 km)	SPAM (Spatial Production Allocation Model) data of the International Food Policy research Institute (IFPRI)[14]
Daily reference evapotranspiration (Eto) data	FAO's AgERA5 dataset[85]
Daily precipitation data	Boogaard et al[86]
Total available water capacity (TAWC)	Fischer et al[87]
Curve number (CN)	Jaafar, Ahmad, & El Beyrouthy[88]

Data	Source
Crop coefficient Kc as well as length of growth stages	Allen, Pereira, Raes, and Smith[89]
Crop calendars	SAGE[90,91], MIRCA2000[92], some additional regional sources[93,94]
Irrigation efficiency (IE)	multiple sources[95–97], including regional/local studies in Algeria[98–101], Botswana[102], Burkina Faso[103], Côte d'Ivoire[104], Egypt[105–107], Ethiopia[107–111], Ghana[112], Kenya[107], Libya[113,114], Malawi[115,116], Mali[117], Morocco[118], Niger[117], South Africa[119], Sudan[107], Tunisia[120] and Uganda[107]
Livestock distribution for six livestock types (buffaloes, cattle, chickens, goats, pigs and sheep) for the years 2020 and 2010, resolution 5 arc minute (about 10 km)	Gridded Livestock of the World or GLW[49,50]
Power plant water demand in Africa according to fuel types, years 2020 and 2010	Gerbens-Leenes et al[41] and own assessment
Mining datasets that provided input to our own developed mining dataset, with 34 main commodities	"Africa Power–Mining Database"[125] of the World Bank, the geospatial mining datasets of the United States Geological Survey (USGS)[126,127]
international GVA database on sub-sectors of the manufacturing industry, INDSTAT, ISIC database (revision 3 and 4)	UNIDO, the United Nations Industrial Development Organisation[19]
four categories of income classifications based on GNI per capita, Atlas method (USD)	World Bank
NASA Black Marble night-time light intensity map, product VNP46A, a dataset at 15 arc second resolution (500 m at the equator)	NASA Black Marble night-time light intensity map[144]
Natural renewable water in spatial resolution 0.5 degrees (50-55 km)	two global models, i.e. WaterGAP2-2e[147] and LPJmL5-7-10-fire[148,149] within ISIMIP (Inter-Sectoral Impact Model Intercomparison Project)

Supplementary Table 2: Calculated national $WW_{connected, yr}$ amounts

Country	Population –GHS 2020	FACTOR$_{municipal}$ (Eq 1)		Physical water loss LOSS$_{municipal}$ (Eq 3)		WW utilities (without losses)	Municipal WW (includes losses) $WW_{national, yr}$ Aquastat[9] or other (inter)national source		% population piped water access[3]	% population piped water access[3]	Piped WW (Eq 4)	
		Value in %	comment	Value in %	comment	Lcd	Mio m^3/year	Latest year	% in 2020	% in latest year	Includes losses Lcd	without losses $WW_{connected, yr}$ Lcd
Algeria	43,429,381	80	No Data, assumption based on other Maghreb countries[11]	27	No Data, assumption based on other Maghreb countries[11]	154 for Oran[63] and 182 for Algiers and suburbs[64]	3,600 (2017)[9]	2017	72	74	320 based on FAO	233 based on FAO. Take values for Oran and Algiers and FAO for remaining connected population
Angola	33,883,351	80	No Data, assumption based on Mozambique[11]	30	value for Luanda province [11]	43 value for Luanda province [11]	320[9]	2005	42	28	162 based on FAO	114 based on FAO, unrealistically high 2005 value compared to IBNET value. Take IBNET 43
Benin	12,762,654	85	value for Cotonou (capital) and suburbs[11], assumed national	21, 30	21 value for Cotonou (capital) and suburbs[11], national 30	38 value for Cotonou [11]	145[9]	2017	39	40	83 based on FAO	58 based on FAO, IBNET Cotonou 38. Comparable amounts, take IBNET value for connected population

Country	Population –GHS 2020	$FACTOR_{municipal}$ (Eq 1)		Physical water loss $LOSS_{municipal}$ (Eq 3)		WW utilities (without losses)	Municipal WW (includes losses) $WW_{national, yr}$ Aquastat[9] or other (inter)national source		% population piped water access[3]	% population piped water access[3]	Piped WW (Eq 4)	
		Value in %	comment	Value in %	comment	Lcd	Mio m^3/year	Latest year	% in 2020	% in latest year	Includes losses Lcd	without losses $WW_{connected, yr}$ Lcd
Botswana	2,542,114	62; 80	62 für Gaborone[11], 80 for rest of country	28	Value for Gaborone[11], assumed national	89 Value for Gaborone[11]	109[9]	2020	91	91	129 based on FAO	93 based on FAO, reliable value, close to IBNET Gaborone value, 83 for Gaborone and 93 for remaining connected population
Burkina Faso	21,553,986	88	Value for Ouagadougou[11], assumed national	15, 30	15 value for Ouagadougou[11], national 30	41 value for Ouagadougou[11]	376[9]	2005	31	23	value 322 based on FAO	value 225 based on FAO unrealistic old 2005 value, IBNET Ouagadougou 41, Sanreports 38. Take IBNET value 41 for connected population
Burundi	12,133,383	80	No Data, assumption	37	Value for Bujumbura[11], assumed national	104 Value for Bujumbura[11]	43[9]	2005	38	21	Based on FAO 71	Based on FAO 45, IBNET Bujumbura 104, take both for relevant region
Cameroon	26,352,924	65	65 für Douala[11], 80 for rest of country	30	30 for Douala[11], assumed national	59 for Douala[11]	247[9]	2005	42	39	Based on FAO 102	Based on FAO 71, old 2005 value. Recent value IBNET 59 for Douala. Comparable amounts, take IBNET 59 for national connected population

**Country**	**Population –GHS 2020**	**$FACTOR_{municipal}$ (Eq 1)**		**Physical water loss $LOSS_{municipal}$ (Eq 3)**		**WW utilities (without losses)**	**Municipal WW (includes losses) $WW_{national, yr}$** Aquastat[9] or other (inter)national source		**% population piped water access[3]**	**% population piped water access[3]**	**Piped WW (Eq 4)**	
		**Value in %**	**comment**	**Value in %**	**comment**	**Lcd**	**Mio m³/year**	**Latest year**	**% in 2020**	**% in latest year**	**Includes losses Lcd**	**without losses $WW_{connected, yr}$ Lcd**
Central African Republic	5,426,695	75	Adapted value for Bangui due to low WW[11], assumed national	39	39 for Bangui[11], assumed national	38 for Bangui[11]	60[9]	2005	14	20	Based on FAO 192	Based on FAO 117, unrealistically high 2005 value compared to IBNET value. Take IBNET 38 for national connected population
Chad	16,635,274	80	No Data, assumption based on Sudan[11]	30	No Data, assumption based on Sudan[11]	No Data	104[9]	2005	17	17	Based on FAO 162	Based on FAO 114, unrealistically high old 2005 value. Take IBNET 38 of Central African Republic
Congo, republic of	4,750,437	81	No Data, assumption based on DRC[11]	31	No Data, assumption based on DRC[11]	No Data	64	2005	55	54	Based on FAO 92	Based on FAO 63, old 2005 value. Similar to IBNET 70 for Kinshasa. Take FAO value.

**Country**	**Population –GHS 2020**	**$FACTOR_{municipal}$ (Eq 1)**		**Physical water loss $LOSS_{municipal}$ (Eq 3)**		**WW utilities (without losses)**	**Municipal WW (includes losses) $WW_{national, yr}$** Aquastat[9] or other (inter)national source		**% population piped water access[3]**	**% population piped water access[3]**	**Piped WW (Eq 4)**	
		**Value in %**	**comment**	**Value in %**	**comment**	**Lcd**	**Mio m^3/year**	**Latest year**	**% in 2020**	**% in latest year**	**Includes losses Lcd**	**without losses $WW_{connected, yr}$ Lcd**
Côte d'Ivoire/Ivory coast	26,831,953	85	85 for SODECI, Utility Profile Société de Distribution d`Eau de Côte d`Ivoire [11], assumed national	18	18 for SODECI [11], assumed national	37 for SODECI (serves about half of population) [11]	320	2015	48	47	Based on FAO 74	Based on FAO 61. Take IBNET amount 37 (for 2014, serving about half of the population) for national connected population
Democratic Republic of the Congo (DRC)	92,499,516	81	81 for Kinshasa[11], assumed national	31	31 for Kinshasa[11], assumed national	70 for Kinshasa[11]	465	2005	35	26	Based on FAO 83	Based on FAO 58, close to IBNET 70 for Kinshasa. Take IBNET 70 for Kinshasa (6.8M connected) and proportionally spread over remaining population (26.8M =0.35*96M-6.8M) so that resulting connected national average is 58. Result 55 for remaining connected population

Country	Population –GHS 2020	FACTOR$_{municipal}$ (Eq 1)		Physical water loss LOSS$_{municipal}$ (Eq 3)		WW utilities (without losses)	Municipal WW (includes losses) WW$_{national, yr}$ Aquastat[9] or other (inter)national source		% population piped water access[3]	% population piped water access[3]	Piped WW (Eq 4)	
		Value in %	comment	Value in %	comment	Lcd	Mio m³/year	Latest year	% in 2020	% in latest year	Includes losses Lcd	without losses WW$_{connected, yr}$ Lcd
Djibouti	1,094,388	80	No Data, assumption based on Egypt[11]	20	No Data, assumption based on Egypt[11]	No Data	16	2005	83	77	Based on FAO 69	Based on FAO 54. Take this value
Egypt	107,373,797	59-90	59-90 for 20 IBNET[11] utilities, national assumed 80	14-24	14-24 for 20 IBNET[11] utilities, national assumed 20	28-404 for 20 IBNET[11] utilities[11], 72.6M people connected, population weighted average 169	10,750	2018	99	99	Based on FAO 281	Based on FAO 225. Take individual IBNET values (72.6M people). Proportionally spread over remaining population (26.8M =0.99*109M-72.6M) so that resulting national average is 225. Result 340 for remaining connected population
Equatorial Guinea	1,595,990	80	No Data, national assumed 80	30	No Data, assumption based on Cameroon[11]	No Data	16	2005	41	22	Based on FAO 210	Based on FAO 147. Acceptable value, similar range as IBNET 183 value for Libreville in neighbouring Gabon. Take 147 for connected population
Eritrea	3,563,738	75	No Data, assumption based on Ethiopia[11]	21, 30	21 value for Asmara[11], national 30	42 value for Asmara[11]	31	2005	51	38	Based on FAO 84	Based on FAO 59. Old year 2005 value. Take IBNET amount 42 for Asmara for total connected population

Country	Population –GHS 2020	$FACTOR_{municipal}$ (Eq 1)		Physical water loss $LOSS_{municipal}$ (Eq 3)		WW utilities (without losses)	Municipal WW (includes losses) $WW_{national, yr}$ Aquastat[9] or other (inter)national source		% population piped water access[3]	% population piped water access[3]	Piped WW (Eq 4)	
		**Value in %**	**comment**	**Value in %**	**comment**	**Lcd**	**Mio m^3/year**	**Latest year**	**% in 2020**	**% in latest year**	**Includes losses Lcd**	**without losses $WW_{connected, yr}$ Lcd**
Eswatini (Swaziland)	1,184,828	67	67 for Utility Profile Swaziland Water Services Corporation (SWSC)[11], assumed national	30	30 for SWSC [11], assumed national	115 for SWSC [11]	41	2005	60	50	Based on FAO 208	Based on FAO 146. Old year 2005 value. Take IBNET amount 115 for total connected population
Ethiopia	117,038,534	50-92	Range 50-92 for 90 IBNET[11] utilities, national assumed 70	7-47	Range 7-47 for 90 IBNET[11] utilities, national assumed 30	9-115 for 90 IBNET[11] utilities, population weighted average 56	810	2005	42	22	Based on FAO 128	Based on FAO 90, old 2005 value, too high compared to IBNET weighted average 56. Take IBNET individual amounts and for remaining connected population 56.
Gabon	2,313,888	70	No Data, assumption	17, 25	17 value for Libreville[11], national 25	183 value for Libreville[11]	85	2005	88	81	Based on FAO 196	Based on FAO 147. Old 2005 value. Take 183 for Libreville (IBNET, 0.95M people connected). Take 183 for total connected population

Country	Population –GHS 2020	$FACTOR_{municipal}$ (Eq 1)		Physical water loss $LOSS_{municipal}$ (Eq 3)		WW utilities (without losses)	Municipal WW (includes losses) $WW_{national, yr}$ Aquastat[9] or other (inter)national source		% population piped water access[3]	% population piped water access[3]	Piped WW (Eq 4)	
		Value in %	comment	Value in %	comment	Lcd	Mio m^3/year	Latest year	% in 2020	% in latest year	Includes losses Lcd	without losses $WW_{connected, yr}$ Lcd
Gambia	2,522,250	85	No Data, assumption based on Senegal[11]	13, 25	13 value for greater Banjul Area[11], national 25	57 for greater Banjul Area[11]	41	2005	78	61	Based on FAO 110	Based on FAO 82. Old 2005 value. Too high compared to IBNET 57. Take IBNET 57 for total connected population
Ghana	32,052,518	80	No Data, assumption	35	35 value for Ghana National Utility[11], assumed national	39 for Ghana National Utility[11]	300	2016	55	44	Based on FAO 64	Based on FAO 41. Amount almost identical to IBNET value for Ghana National Utility[11], which serves about 18 M people. Take IBNET amount of 39 for total connected population
Guinea	13,218,338	85	National same as Senegal and Côte d'Ivoire	23	23 value for urban centers (Conakry Kindia Mamou)[11], assumed national	29 for urban centers (Conakry Kindia Mamou)[11]	230	2005	23	24	Based on FAO 284	Based on FAO 219. Unrealistic high value compared to IBNET amount 29 for urban centers. Take IBNET value 29 for total connected population
Guinea-Bissau	1,993,918	85	No Data, assumption based on Guinea[11]	34	34 value for city of Bissau[11], assumed national	161 for city of Bissau[11]	34	2005	46	20	Based on FAO 337	Based on FAO 222. Old 2005 value. Too high compared to IBNET 161 for city of Bissau. Take IBNET 161 value for total connected population

Country	Population –-GHS 2020	FACTOR$_{municipal}$ (Eq 1)		Physical water loss LOSS$_{municipal}$ (Eq 3)		WW utilities (without losses)	Municipal WW (includes losses) WW$_{national, yr}$ Aquastat[9] or other (inter)national source		% population piped water access[3]	% population piped water access[3]	Piped WW (Eq 4)	
		Value in %	comment	Value in %	comment	Lcd	Mio m³/year	Latest year	% in 2020	% in latest year	Includes losses Lcd	without losses WW$_{connected, yr}$ Lcd
Kenya	52,262,592	37-98	Range 37-98 for 103 IBNET[11] utilities, national assumed 75	7-55	Range 7-55 for 103 IBNET[11] utilities, national assumed 30	7-202 for 103 IBNET[11] utilities, population weighted average 57	495	2016	33	33	Based on FAO 85	Based on FAO 60. Amount almost identical to IBNET population-weighted value of 57 for 103 utilities[11]. Take IBNET individual amounts and for remaining connected population 57.
Lesotho	2,225,949	65	No Data, assumption based on South Africa/Eswatini[11]	30	30 value for city of Maseru[11], assumed national	90 for city of Maseru[11]	20	2005	71	63	Based on FAO 45	Based on FAO 31. Take IBNET 90 for city of Maseru[11] and for remaining connected population 31
Liberia	5,095,665	85	National same as Côte d'Ivoire	37	37 value for Liberia Water and Sewer Corporation [11], assumed national	6 for Liberia Water and Sewer Corporation[11]	80	2005	6	10	Based on FAO 664	Based on FAO 418. Unrealistic high value for the year 2005. Take IBNET amount 6 for total connected population

Country	Population –GHS 2020	FACTOR$_{municipal}$ (Eq 1)		Physical water loss LOSS$_{municipal}$ (Eq 3)		WW utilities (without losses)	Municipal WW (includes losses) WW$_{national, yr}$ Aquastat[9] or other (inter)national source		% population piped water access[3]	% population piped water access[3]	Piped WW (Eq 4)	
		Value in %	comment	Value in %	comment	Lcd	Mio m^3/year	Latest year	% in 2020	% in latest year	Includes losses Lcd	without losses WW$_{connected, yr}$ Lcd
Libya	6,651,786	81	81 for Utility Tripoli, assumed national	26	26 for Utility Tripoli, assumed national	250 for Utility Tripoli	700	2010	78	70	Based on FAO 422	Based on FAO 312. Value comparable with IBNET 250 for Utility Tripoli. FAO value of 2010, IBNET value more recent. Take IBNET 250 for all connected population.
Madagascar	28,225,171	83	83 for utility Jiro sy Rano Malagasy [11], assumed national	33	33 for utility Jiro sy Rano Malagasy[11], assumed national	58 for utility Jiro sy Rano Malagasy[11]	395	2007	35	28	Based on FAO 190	Based on FAO 127. Too high value for the year 2007 compared to IBNET 58 for utility Jiro sy Rano Malagasy[11]. Take IBNET 58 for all connected population.
Malawi	19,290,056	54-76	Range 54-76 for 5 IBNET[11] utilities, national assumed 70	14-41	Range 14-41 for 5 IBNET[11] utilities, national assumed 30	40-74 for 5 IBNET[11] utilities, population weighted average 56	143	2005	21	22	Based on FAO 139	Based on FAO 97. Too high value from 2005 as compared to IBNET population weighted average 56. Take IBNET individual amounts and for remaining connected population 56

Country	Population –GHS 2020	$FACTOR_{municipal}$ (Eq 1)		Physical water loss $LOSS_{municipal}$ (Eq 3)		WW utilities (without losses)	Municipal WW (includes losses) $WW_{national, yr}$ Aquastat[9] or other (inter)national source		% population piped water access[3]	% population piped water access[3]	Piped WW (Eq 4)	
		Value in %	comment	Value in %	comment	Lcd	Mio m^3/year	Latest year	% in 2020	% in latest year	Includes losses Lcd	without losses $WW_{connected, yr}$ Lcd
Mali	21,205,136	78	78 for utility Bamako[11], assumed national	29	29 for utility Bamako[11], assumed national	75 for utility Bamako[11]	107	2005	50	28	Based on FAO 77	Based on FAO 55. Take IBNET 75 for utility Bamako[11] (2.6M people connected). Proportionally spread over remaining population (8M =0.5*21.2M-2.6M) so that resulting national average is 55. Result 48 for remaining connected population
Mauritania	4,575,381	50	50 for utility Mauritania Société Nationale des Eaux[11], national assumed 75	28	28 for utility Mauritania Société Nationale des Eaux[11], assumed national	125 for utility Mauritania Société Nationale des Eaux[11]	95	2005	58	34	Based on FAO 260	Based on FAO 188. Too high value from 2005 as compared to IBNET 125 for utility Mauritania Société Nationale des Eaux[11]. Take 125 for connected population. Result 48 for remaining connected population

Country	Population –GHS 2020	FACTOR$_{municipal}$ (Eq 1)		Physical water loss LOSS$_{municipal}$ (Eq 3)		WW utilities (without losses)	Municipal WW (includes losses) WW$_{national, yr}$ Aquastat[9] or other (inter)national source		% population piped water access[3]	% population piped water access[3]	Piped WW (Eq 4)	
		Value in %	comment	Value in %	comment	Lcd	Mio m^3/year	Latest year	% in 2020	% in latest year	Includes losses Lcd	without losses WW$_{connected, yr}$ Lcd
Morocco (including Western Sahara)	36,853,638	80-92	Range 80-92 for 12 utilities[65–74], national assumed 80	16-33	Range 16-33 for 12 utilities, national assumed 25	61-229 for 12 utilities[65–74], population weighted average 118	1,063	2010	78	70	Based on FAO 129	Based on FAO 96, close to IBNET average 118. Take IBNET values (13.4M people). Proportionally spread over remaining population (15.3M =0.78*36.8M-13.4M) so that resulting connected national average is 96. Result 77 for remaining (non-IBNET) connected population
Mozambique	31,284,630	62-88	Range 62-88 for 18 IBNET[11] utilities, national assumed 80	14-34	Range 14-34 for 18 IBNET[11] utilities, national assumed 25	33-87 for 18 IBNET[11] utilities, population weighted average 67	372	2015	39	34	Based on FAO 113	Based on FAO 85, close to IBNET average 67. Take individual IBNET values and 67 for remaining connected population
Namibia	2,394,088	80	80 assumed national	10-16	Range 10-16 for 3 IBNET[11] utilities, national assumed 15	91-219 for 3 IBNET[11] utilities including Windhoek, population weighted average 198	73	2005	83	73	Based on FAO 139	Based on FAO 118, old value from 2005. Take IBNET individual values and for remaining connected population IBNET weighted average 198

Country	Population –-GHS 2020	FACTOR$_{municipal}$ (Eq 1)		Physical water loss LOSS$_{municipal}$ (Eq 3)		WW utilities (without losses)	Municipal WW (includes losses) WW$_{national, yr}$ Aquastat[9] or other (inter)national source		% population piped water access[3]	% population piped water access[3]	Piped WW (Eq 4)	
		Value in %	comment	Value in %	comment	Lcd	Mio m^3/year	Latest year	% in 2020	% in latest year	Includes losses Lcd	without losses WW$_{connected, yr}$ Lcd
Niger	24,445,509	82	82 for Utility Profile Societe De Patrimoine Des Eaux Du Niger (Niamey)[11], assumed national	12, 30	12 for Utility Profile Societe De Patrimoine Des Eaux Du Niger (Niamey)[11], 30 assumed national	63 for Niamey	193	2019	39	38	Based on FAO 61	Based on FAO 49, recent value (year 2019) close to IBNET value for Niamey 63. Take latter for Niamey (2.6M people connected). Proportionally spread over remaining population (7M =0.39*24.5M-2.6M) so that resulting connected national average is 49. Result 44 for remaining (non-IBNET) connected population
Nigeria	208,056,607	55-99	Range 55-99 for 37 IBNET[11] utilities, national assumed 80	6-75	Range 6-75 for 37 IBNET[11] utilities, national assumed 35	1-329 for 37 IBNET[11] utilities. Population weighted average 30	5,000	2010	28[11] (instead of 13[3])	28[11]	Based on FAO 235	Based on FAO 153, in range of IBNET values, but too high according to population weighted IBNET average 30. Take latter for non-IBNET connected population

Country	Population –GHS 2020	FACTOR$_{municipal}$ (Eq 1)		Physical water loss LOSS$_{municipal}$ (Eq 3)		WW utilities (without losses)	Municipal WW (includes losses) WW$_{national, yr}$ Aquastat[9] or other (inter)national source		% population piped water access[3]	% population piped water access[3]	Piped WW (Eq 4)	
		**Value in %**	**comment**	**Value in %**	**comment**	**Lcd**	**Mio m³/year**	**Latest year**	**% in 2020**	**% in latest year**	**Includes losses Lcd**	**without losses WW$_{connected, yr}$ Lcd**
Rwanda	13,806,452	75	75 assumed national	27	27 value for Kigali[11], assumed national	65 value for Kigali[11]	230	2019	44	44	Based on FAO 112	Based on FAO 82. Recent value (year 2019) close to IBNET 65 value for Kigali. Take IBNET value for Kigali (0.7M people connected). Proportionally spread over remaining population (5.3M =0.44*13.8M-0.7M) so that resulting connected national average is 82. Result 84 for remaining (non-IBNET) connected population
Senegal	16,447,176	92, 85	92 value for Dakar[11], national 85	15, 25	15 value for Dakar[11], national 25	59 value for Dakar[11]	261	2017	75	72	Based on FAO 64	Based on FAO 48. Recent value close to IBNET 59 value for Dakar. Take IBNET value for Dakar (5.8M people connected). Proportionally spread over remaining population (5.3M =0.75*16.5-5.8M) so that resulting connected national average is 48. Result 38 for remaining (non-IBNET) connected population

Country	Population –-GHS 2020	FACTOR$_{municipal}$ (Eq 1)		Physical water loss LOSS$_{municipal}$ (Eq 3)		WW utilities (without losses)	Municipal WW (includes losses) WW$_{national, yr}$ Aquastat[9] or other (inter)national source		% population piped water access[3]	% population piped water access[3]	Piped WW (Eq 4)	
		Value in %	comment	Value in %	comment	Lcd	Mio m^3/year	Latest year	% in 2020	% in latest year	Includes losses Lcd	without losses WW$_{connected, yr}$ Lcd
Sierra Leone	8,228,113	85	No data, national same as Liberia	37	No data, national same as Liberia	No data	111	2005	21	25	Based on FAO 221	Based on FAO 139. Unrealistic high value from the year 2005. Take IBNET value 29 of urban centres of neighbouring Guinea
Somalia	16,629,887	70	No data, national same as Ethiopia	30	No data, national same as Ethiopia	No data	15	2005	47	18	Based on FAO 21	Based on FAO 15. Old value from 2005. Take average IBNET value 56 of neighbouring Ethiopia
South Africa	58,836,310	54-97	Range 54-97 for 24 IBNET[11] utilities, national assumed 70	8-45	Range 8-45 for 24 IBNET[11] utilities, national 33[60]	115-411 for 24 IBNET[11] utilities, with 24M connected people, population weighted average 196	4,282, national report[60]	2020	90	90	Based on national report[60] 215	Based on national report[60] 144, close to IBNET population weighted average 196. Take IBNET value for IBNET utilities (24M people connected). Proportionally spread over non-IBNET population (29M =0.90*58.8-24M) so that resulting connected national average is 144. Result 101 for remaining (non-IBNET) connected population
South Sudan	10,490,534	85	No data, national same as Sudan	25	No data, national same as Sudan	No data	193	2012	5	7	Based on FAO 702	Based on FAO 527. Unrealistic high value. Take FAO value 129 of Sudan

Country	Population –GHS 2020	FACTOR$_{municipal}$ (Eq 1)		Physical water loss LOSS$_{municipal}$ (Eq 3)		WW utilities (without losses)	Municipal WW (includes losses) WW$_{national, yr}$ Aquastat[9] or other (inter)national source		% population piped water access[3]	% population piped water access[3]	Piped WW (Eq 4)	
		Value in %	comment	Value in %	comment	Lcd	Mio m³/year	Latest year	% in 2020	% in latest year	Includes losses Lcd	without losses WW$_{connected, yr}$ Lcd
Sudan	44,430,364	82-97	Range 82-97 for 5 IBNET[11] utilities, national assumed 85	4-41	Range 4-41 for 5 IBNET[11] utilities, national assumed 30	36-274 for 5 IBNET[11] utilities, including Khartoum, population weighted average 241	950	2012	56	41	Based on FAO 172	Based on FAO 129, in range of IBNET. Take IBNET values (4.2M people connected). Proportionally spread over non-IBNET population (20.7M =0.56*44.4-4.2M) so that resulting connected national average is 129. Result 106 for remaining (non-IBNET) connected population
Togo	8,580,329	79	79 value for SODECA, Utility Profile Societe Togolaise des Eaux (Lome)[11], assumed national	10, 25	10 value for SODECA Lome[11], national 25	40 for SODECA Lome[11]	141	2005	31	31	213	Based on FAO 160. Old value from 2005, unrealistically high compared to IBNET 40 for SODECA Lome[11]. Take IBNET 40 for all connected population

Country	Population –GHS 2020	FACTOR$_{municipal}$ (Eq 1)		Physical water loss LOSS$_{municipal}$ (Eq 3)		WW utilities (without losses)	Municipal WW (includes losses) WW$_{national, yr}$ Aquastat[9] or other (inter)national source		% population piped water access[3]	% population piped water access[3]	Piped WW (Eq 4)	
		Value in %	comment	Value in %	comment	Lcd	Mio m^3/year	Latest year	% in 2020	% in latest year	Includes losses Lcd	without losses WW$_{connected, yr}$ Lcd
Tunisia	12,182,900	81-90	Range 81-90 for 4 utilities, national assumed 85	25	25 for 4 utilities, national assumed 25	119-136 for 4 utilities, population weighted average 124	816	2020	92	92	203	Based on FAO 152. IBNET average value total connected national population 120. Take individual IBNET values.
Uganda	44,251,647	57-95	Range 57-95 for 7 IBNET[11] utilities, national assumed 85	20-41	Range 20-41 for 7 IBNET[11] utilities, national assumed 30	10-15 for 7 IBNET[11] utilities, population weighted average 14	328	2008	23	16	184	Based on FAO 129. Old value from 2008, unrealistically high compared to IBNET population-weighted average of 14[11]. Take IBNET values and 14 for remaining connected population
United Republic of Tanzania	61,736,013	75	No Data, assumption based on Kenya[11]	13-38	Range 13-38 for 27 IBNET[11] utilities, national assumed 30	17-74 for 27 IBNET[11] utilities, 13M connected people, population weighted average 46	527	2005	40	35	105	Based on FAO 74. Old value of 2005. Take individual IBNET values and IBNET average 46 for remaining connected population

Country	Population –GHS 2020	FACTOR$_{municipal}$ (Eq 1)		Physical water loss LOSS$_{municipal}$ (Eq 3)		WW utilities (without losses)	Municipal WW (includes losses) WW$_{national, yr}$ Aquastat[9] or other (inter)national source		% population piped water access[3]	% population piped water access[3]	Piped WW (Eq 4)	
		Value in %	comment	Value in %	comment	Lcd	Mio m³/year	Latest year	% in 2020	% in latest year	Includes losses Lcd	without losses WW$_{connected, yr}$ Lcd
Zambia	19,282,069	55-88	Range 55-88 for 12 IBNET[11] utilities, national assumed 70	27-56	Range 27-56 for 12 IBNET[11] utilities, national assumed 35	25-178 for 12 IBNET[11] utilities, 6.8M connected people, population weighted average 67	290	2005	34	32	212	Based on FAO 138. Old value from 2005, unrealistically high compared to IBNET population-weighted average of 67[11]. Take individual IBNET values and IBNET average 67 for remaining connected population
Zimbabwe	15,703,255	51-98	Range 51-98 for 31 IBNET[11] utilities, national assumed 75	9-56	Range 9-56 for 31 IBNET[11] utilities, national assumed 35	15-253 for 31 IBNET[11] utilities, , 3.8M connected people, population weighted average 124	649	2018	28	29	408	Based on FAO 265. Unrealistically high compared to IBNET population-weighted average of 124[11]. Take individual IBNET values and IBNET average 124 for remaining connected population

2. **Comparison WD years 2020 and 2010**

Supplementary Table 3: Comparison crop specific irrigation WD amounts 2020 and 2010

Crop group	crop	Amount (Mm^3) in 2020	Amount (Mm^3) in 2010	Change in Mm^3	Change from 2010 (%)
cereals	wheat	21,170	21,035	135	1
cereals	maize	19,910	17,042	2,868	17
cereals	rice	39,136	28,882	10,254	36
cereals	paddy	16,210	11,117	5,093	46
cereals	Sum: rice+paddy	55,345	39,999	15,347	38
cereals	sorghum	4,679	5,899	-1,220	-21
cereals	barley	1,449	2,400	-951	-40
cereals	millet	1	3	-1	-57
cereals	pearl millet	219	367	-148	-40
cereals	other cereals	711	731	-20	-3
**cereals**	**SUM**	**103,484**	**87,475**	**16,010**	**18**
roots and tubers	potato	5,620	4,325	1,296	30
roots and tubers	cassava	10	7	3	48
roots and tubers	sweet potato	130	98	32	32
roots and tubers	yams	0	0	0	0
roots and tubers	other roots	25	34	-9	-27
**roots and tubers**	**SUM**	**5,785**	**4,464**	**1,322**	**30**
fruit	banana	4,826	2,932	1,894	65
fruit	plantain	0	0	0	0
fruit	citrus	5,073	No separate Data in SPAM2010 - included in "other tropical fruit"		
fruit	other tropical fruit	14,945			
fruit	other tropical fruit incl citrus	20,018	16,988	3,030	18

Crop group	crop	Amount (Mm^3) in 2020	Amount (Mm^3) in 2010	Change in Mm^3	Change from 2010 (%)
fruit	temperate fruit	10,334	8,472	1,861	22
**fruit**	**SUM**	**35,178**	**28,392**	**6,786**	**24**
vegetables	onions	4,440	No separate Data in SPAM2010 - only "vegetables"		
vegetables	tomatoes	4,790			
vegetables	other vegetables	10,099			
**vegetables**	**SUM**	**19,329**	**9,006**	**10,323**	**115**
pulses	beans	417	383	34	9
pulses	chickpea	26	42	-16	-38
pulses	cowpea	14	16	-2	-15
pulses	pigeonpeas	0	0	0	0
pulses	lentil	46	60	-14	-23
pulses	other pulses	593	947	-354	-37
**pulses**	**SUM**	**1,096**	**1,449**	**-352**	**-24**
oilcrops	coconut	0	0	0	0
oilcrops	groundnut	7,457	3,803	3,654	96
oilcrops	oil palm	75	70	5	7
oilcrops	rapeseed	0	0	0	0
oilcrops	sesame seed	192	216	-24	-11
oilcrops	soybeans	1,246	401	845	211
oilcrops	sunflower	797	631	166	26
oilcrops	other oil crops	7,345	8,240	-895	-11
**oilcrops**	**SUM**	**17,112**	**13,360**	**3,752**	**28**
sugar crops	sugarbeet	3,058	2,374	684	29
sugar crops	sugarcane	22,677	19,433	3,244	17
**sugar crops**	**SUM**	**25,735**	**21,807**	**3,928**	**18**
stimulants	arabic coffee	883	812	70	9
stimulants	robust coffee	0	0	0	0
stimulants	tea	384	190	193	102
stimulants	cocoa	0	0	0	0

Crop group	crop	Amount (Mm^3) in 2020	Amount (Mm^3) in 2010	Change in Mm^3	Change from 2010 (%)
stimulants	tobacco	342	421	-80	-19
**stimulants**	**SUM**	**1,608**	**1,424**	**184**	**13**
fibres	cotton	5,880	4,139	1,741	42
fibres	other fibre crops	198	214	-16	-7
**fibres**	**SUM**	**6,079**	**4,353**	**1,726**	**40**
rest of crops	rubber	3	No separate Data in SPAM2010 - only "rest of crops"	3	
rest of crops	other rest of crops	9,765			
**rest of crops**	**SUM**	**9,768**	**7,824**	**1,944**	**25**
**all crops**	**SUM**	**225,174**	**179,552**	**45,622**	**25**

3 Validation

In this section we validate our results by 1) comparing them with data from Aquastat[9]; 2) comparing them with the few studies that provide spatially distributed sectoral water demand data covering the whole of Africa and 3) comparing them with other modelling approaches. The results are listed in Supplementary Tables 4 to 10 and figures 1 to 5.

We first compare our results with data from Aquastat[9], which provides only national data, not spatially distributed data (Supplementary Table 4).

Relevant spatially distributed assessments with which we compare our results are:

- Florke et al[13], who quantified municipal and industrial WDs for the year 2010 on a global level in 5 arc-minutes (approximately 10 km), yet do not provide their data open access (Supplementary Table 5);
- Huang et al[12], who quantified sectoral WDs for the year 2010 on a global level in a spatial resolution of 0.5 degrees (50-55 km) and provide their results open access (Supplementary Table 6);
- ISIMIP[158,159], who quantified sectoral WDs for the years 2010 and 2020 on a global level in a spatial resolution of 0.5 degrees and provide their results open access (Supplementary Table 7);
- Panda and Kim[160] who quantified industrial WDs for the years 2005, 2010 and 2015 on a global level in a spatial resolution of 0.5 degrees and provide their results open access. We compare with our year 2010 results (Supplementary Table 8 and 9);

Aquastat provides an overall African water withdrawal (WW) amount of 236,029 Mm^3 for 2020, whereas we compute a WD of 347,033 Mm^3 (Supplementary Table 5). Part of this difference is due to WDs not being met, part due to certain countries not reporting or not having the capacity to report water withdrawal amounts. For municipal WD, we compute a substantial higher amount (59,796 Mm^3), as non-piped water access is lacking for 717 people with a total WD of 23,989 Mm^3, which Aquastat does not account for. Our assessment of 35,807 Mm^3 for piped access is quite similar to Aquastat's amount of 34,562 Mm^3 though.

Also for other sectors we compute a higher WD compared to the WW registered in official Aquastat statistics. For industry, which comprises manufacturing industry, cooling water for electricity (without hydropower) as well as mining, Aquastat's continental value is 14,808 Mm^3.

We compute 28,322 Mm^3 (without hydropower), of which manufacturing industry accounts for 15,021 Mm^3, electricity for 10,053 Mm^3 and mining for 3,247 Mm^3. Our total amount is in line with ISIMIP[158,159], who compute 30,202 Mm^3 for the year 2020. Aquastat does not provide these statistics for African countries on manufacturing industry, electricity or mining, only for total industry. As an example, only for Zimbabwe Aquastat provides a statistic on electricity. i.e., 48 Mm^3 for the year 2020, which is a statistic interpolated from the year 2015. Our study quantifies a WW of 15 Mm^3 for cooling of thermoelectric plants, based on 15 power plants. For livestock, Aquastat's value is much lower (2,097 Mm^3) than the value we compute (10,706 Mm^3). Aquastat's value appears to be an underestimation, potentially based on missing data, as our value is much more in line with Huang et al[12], who compute 12,212 Mm^3 for the year 2010. We compute 8,186 Mm^3 for the year 2010.

For the year 2000, where livestock numbers were considerably lower throughout Africa, Mekonnen and Hoekstra[121] compute an amount 5,015 Mm^3. This is considerably higher than the Aquastat amount for 2020, confirming the latter is an underestimation, especially because livestock numbers have increased substantially from 2000 to 2020. Aquastat reports 184,562 Mm^3 for irrigation without reporting values for different crops, whereas we quantify 225,174 Mm^3 wile differentiating between 46 crops.

Supplementary Table 4: Comparison of our results with Aquastat statistics

**Study/ source**	**Sector**	**Year**	**Spatial resolution**	**Mm^3**	**Open access**	**Our study**
Aquastat [9]	All sectors	2020	National data	236,029	yes	347,033 Mm^3
	Municipal – only piped access			34,562		59,796 Mm^3, of which 35,807 Mm^3 piped access, 23,989 Mm^3 non-piped access
	Irrigation<br>No differentiation different crops			184,562		225,174 Mm^3
	Livestock<br>No differentiation livestock classes			2,097		10,706

Study/ source	Sector	Year	Spatial resolutio n	Mm^3	Open access	Our study
	industry, which comprises manufacturing industry, cooling water for electricity (without hydropower) and mining. No differentiation between manufacturing industry subsectors, electricity fuel types or mining commodities			14,808, no details on sectors (man industry, electricity, mining)		28,322 Mm^3 (without hydropower), with manufacturing industry 15,021 Mm^3, electricity 10,053 Mm^3 and mining 3,247 Mm^3

Florke et al[13] published an influential and much cited study in 2013 on domestic and industrial water uses of the past 60 years, up to the year 2010 (Supplementary Table 5). This study has a detailed spatial resolution of 5 arc-minutes (approximately 10 km). We provide our results open access whereas Florke et al[13] do not. We can therefore not compare results on the national or grid level. We also provide recent results for 2020, whereas the most recent year of Florke et al[13] is 2010.

The authors report for municipal piped water use 21,779 Mm^3 for Africa, an amount comparable to our value 25,700 Mm^3. However, where we use the actual data of +450 water supply utilities in downscaling national amounts over a 2010 population raster, Florke et al[13] do not use individual water supply utility data for Africa and distribute national results over a population raster. We compute municipal WD in higher spatial resolution (2,5 arc-minutes (approximately 5 km)). We compute a non-piped WD of 20,749 Mm^3 whereas Florke et al[13] do not.

Florke et al[13] compute 15,248 Mm^3 for manufacturing industry, a value higher than the amount for 2010 we compute (11,856 Mm^3), although in the same range. The authors do not account for subsectors in manufacturing industry and we compute municipal WD in higher spatial resolution (2,5 arc-minutes (approximately 5 km)), spatially distributed according to the NASA Black Marble night-time light intensity map[144].

For electricity (without hydropower), Florke et al[13] compute 21,739 Mm^3, whereas we compute 5,006 Mm^3. This is a substantial difference, where the value of Florke et al[13] seems to be on the high side. Huang et al[12] compute 5,867 Mm^3 for the year 2010 for Africa and Sanchez et al[161] compute 5,418 Mm^3 for the year 2016, amounts that are in our range. Also, Florke et al[13] used the World Electric Power Plants Data Set of the Utility Data Institute for the spatial distribution of

electricity water use, a database now managed as commercial product by the private company S&P Global Platts. It is therefore not open-access available and data computed with this database cannot be published open-access. We generated our power plant database with related WD for 2010 (1,511 power plants) and 2020 (2,357 power plants) ourselves and provide it open access

Supplementary Table 5: Comparison of our results with Florke et al[13]

Study/ source	Sector	Year	Spatial resolution	Mm^3	Open access	Our study
Florke et al[13]	Municipal<br>National domestic piped water use is downscaled to grid cells within the country based on population density maps<br>No water supply utility data are accounted for	2010 most recent year	5 arc-minutes, approximately 10 km	21,779	no	25,700 Mm^3 piped access, 20,749 Mm^3 non-piped access
	Manufacturing industry<br>No differentiation between manufacturing industry subsectors<br>National water use is allocated to grid cells according to the distribution of urban population			15,248, largest part in Northern Africa 10,464		11,856
	Electricity for cooling<br>No hydropower<br>World Electric Power Plants Data Set of the Utility Data Institute used, last version 2010			21,739, largest part in Northern Africa 16,707		5,006 without hydropower

Huang et al[12] published in 2018 a study on global gridded sectoral water withdrawals for 1971–2010, in a spatial resolution of 0.5 degrees (50-55 km) including the same 6 sectors as our study (Supplementary Table 6). Their results are published open-access, which provides the opportunity to compare their and our results on the national or grid level for the year 2010, their most recent assessment.

Supplementary Table 6: Comparison of our results with Huang et al[12]

Study/ source	Sector	Year	Spatial resolution	Mm^3	Open access	Our study
Huang et al[12]	Same 6 sectors as our study<br>Sectoral national withdrawal data based on Aquastat[9], apart from USA<br>For electricity, manufacturing industry and mining:<br>population density maps for the spatial downscaling	2010, most recent year	0.5 degrees, i.e. 50-55 km	253,261-271,843	yes	274,763 (including non-piped municipal WD)
	Municipal – only piped access			32,177		25,700 Mm^3 piped access, 20,749 Mm^3 non-piped access
	Irrigation			195,333-213,915		179,552
	Livestock<br>GWL data for the year 2005 used			12,394		8,186<br>GWL data for the year 2010 used
	Electricity, without hydropower			5,949		5,006 without hydropower
	Manufacturing industry<br>population density to downscale water withdrawal			6,315		11,856
	Mining<br>population density to downscale water withdrawal			1,093		1,964

Our total result (274,763 Mm^3) is quite similar to theirs (253,261-271,843 Mm^3), while the sectoral Africa-wide results are also in the same value range. We compute 25,700 Mm^3 for piped municipal WD, whereas Huang et al[12] compute 32,177 Mm^3. They do not compute non-piped access WD. We compute 179,552 Mm^3 for irrigation, they 195,333-213,915 Mm^3. For livestock the values are 12,394 Mm^3 and 8,186 Mm^3, respectively. For electricity (without hydropower) they are 5,949 Mm^3 and 5,006 Mm^3, very similar amounts. For manufacturing industry, they are 6,315 Mm^3 and 11,856 Mm^3, while for mining the values are 1,093 Mm^3 and 1,964 Mm^3, respectively.

There are however large differences between the two studies.

First, our study has a spatial detail incomparable with Huang et al[12]. We compute the sectors municipal WD and manufacturing WD in a spatial resolution of 2,5 arc minutes (about 5 km or 0.041667 degrees), while their resolution is 0.5 degrees. This means that for each grid cell they compute, we quantify 144 grid cells. For irrigation and livestock, we compute WDs in 5 arc minutes (about 5 km or 0.083333 degrees). For each grid cell they compute, we quantify 36 grid cells. For electricity, Huang et al[12] downscale national amounts according to population density, whereas we compiled an inventory of 1,511 power plants differentiating between fuel types for the year 2010 (and 2,357 power plants in 2020). For mining, Huang et al[12] downscale national amounts according to population density, whereas we compiled an inventory of 385 mines differentiating between commodities for the year 2010 (and 682 mines in 2020). Power plants and mines are often is sparsely located areas, with a high local WD, representing often the highest WD in a certain region/(sub)basin (Figure 5 in main text). Huang et al[12] dilute these high local WDs over a spatially dispersed population raster. This has a substantial influence on the spatial distribution of total WDs and associated water scarcity.

Second, we account for multiple subsectors (crops, livestock classes, electricity fuel types, manufacturing industries, mining commodities), while Huang et al[12] do not. We also account for hydropower. Third, we account for the actual data of +450 water supply utilities in downscaling national municipal WD amounts over a 2010 population raster, while Huang et al[12] only use national Aquastat amounts. Fourth, the most recent year of Huang et al[12] is 2010, whereas we compute 2010 and 2020.

We compare the total WD between our study and Huang et al for the year 2010 on the grid level in Supplementary Figure 1. Although the sectors municipal WD and manufacturing WD are generated in a spatial resolution of 2,5 arc minutes (0.041667 degrees), the resolution of our total WD is computed in 5 arc minutes (0.08333 degrees)(Supplementary Figure 1A). Compared to the total WD computed by Huang et al (Supplementary Figure 1C), generated in 0.5 degrees, we thus compute 36 grid cells for each Huang et al grid cell. This difference is clearly visible between the two maps. To be able to compare both results directly at the grid level, we aggregate our results to 0.5 degrees (Supplementary Figure 1B). A comparison of national amounts shows a high correlation (R^2=0.95) and a median of differences (MoD) relative to our study of -25% (Supplementary Figure 1D). On the individual grid level at 0.5 degrees resolution, the correlation is lower at 0.78% and the MoD is -5% (Supplementary Figure 1E).

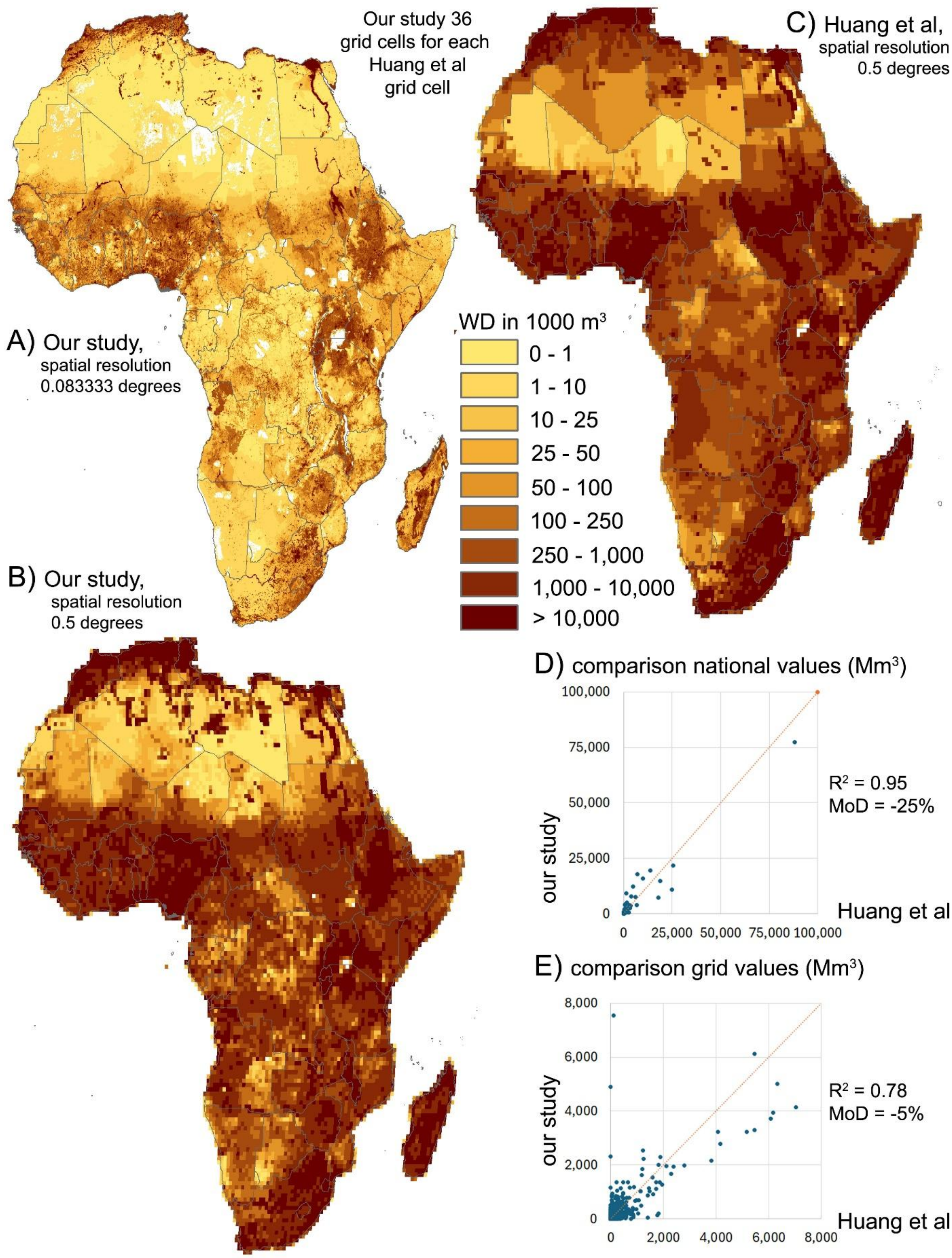

Supplementary Figure 1: Comparison total WD between our study and Huang et al for the year 2010. A) Our study in resolution 0.083333 degrees (5 arc minute, 10 km); B) our study aggregated to 0.5 degrees; C) Huang et al; D) comparison national amounts with R^2 spatial correlation and MoD Median of Differences relative to our study, Red line is 1:1 gradient; E) comparison grid amounts for spatial resolution 0.5 degrees

To compare the two analyses on the sectoral level, we choose electricity WD without hydropower, for which we compute 5,006 Mm^3 and Huang et al 5,949 Mm^3. Excluding hydropower, we include 1,127 power plants, differentiated by fuel types (Supplementary Figure 2A). All power plants have been double-checked on location by means of satellite images, providing the best and most localized power plant database available for Africa. Transforming and aggregating this dataset into a 0.5 degrees grid results in 763 grid cells (Supplementary Figure 2B), thus already losing a vast amount of exact spatial information on individual power plants. Comparing latter grid with the 0.5 degrees grid of Huang et al (Supplementary Figure 2C, downscaled by population density), shows that only 3,187 Mm^3 from a total of 5,949 Mm^3 of Huang et al are covered by our 763 grid cells. This means that the remaining 2,762 Mm^3 have been spatially distributed over population grid cells where in reality there are no power plants. This shows the vast superiority in spatial detail of our study. Even in the 763 grid cells of our study, correlation is rather low (R^2=0.62) and MoD with +7950 very high, indicating large differences between grid cell amounts (Supplementary Figure 2D).

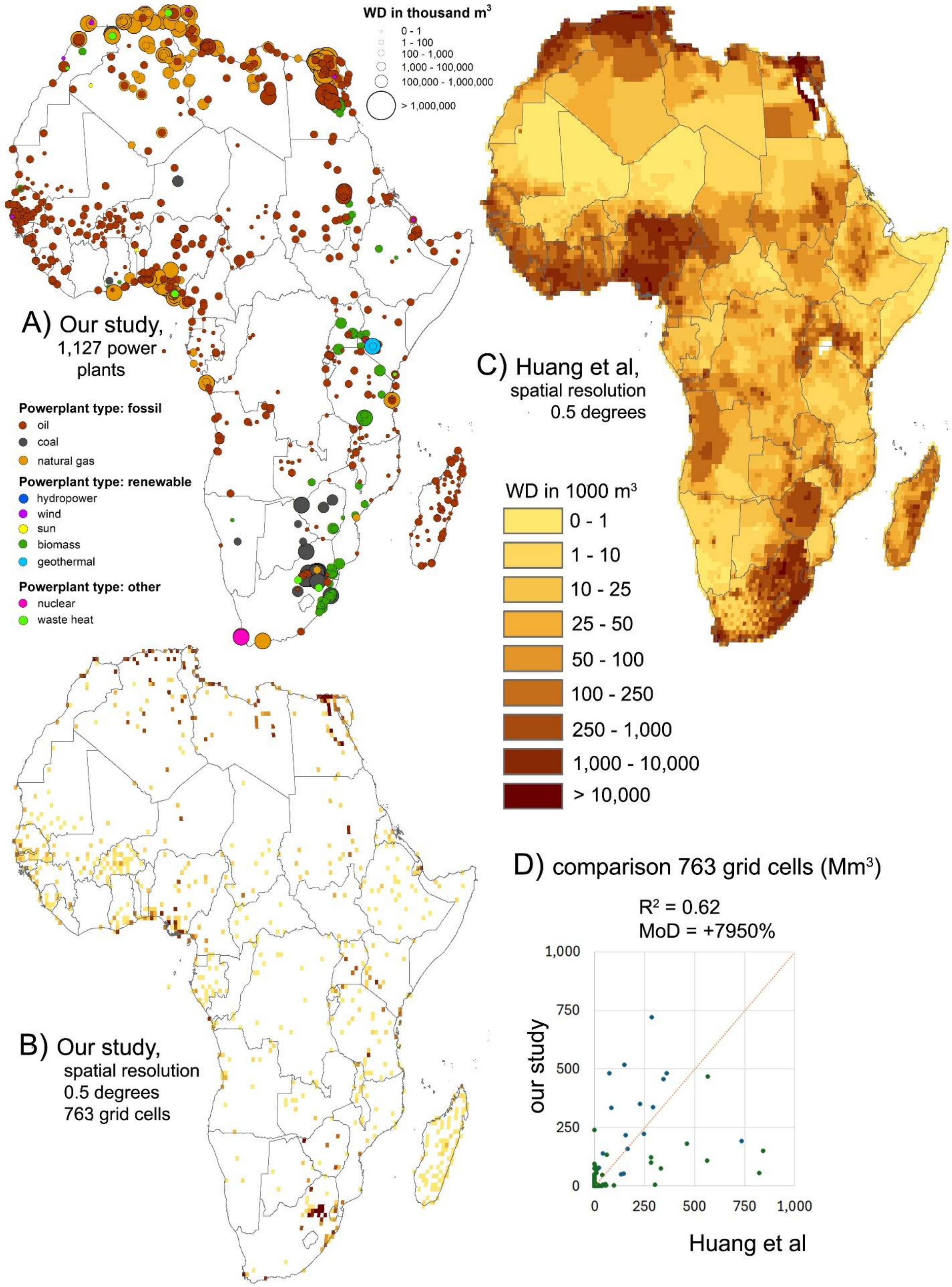

Supplementary Figure 2: Comparison electricity (without hydropower) WD between our study and Huang et al for the year 2010. A) Our 1,127 power plants; B) our study aggregated to 0.5 degrees; C) Huang et al; D) comparison grid amounts with R^2 spatial correlation and MoD (Median of Differences) relative to our study

ISIMIP[158,159], the Inter-Sectoral Impact Model Intercomparison Project, also provides sectoral water withdrawal data for the years 2020 and 2010 on municipal and industrial WD, in a spatial resolution of 0.5 degrees (50-55 km)(Supplementary Table 7). ISIMIP's results are published open-access, which provides the opportunity to compare ISIMIP's and our results on the national or grid level.

Our municipal WDs (35,807 Mm^3 for 2020 and 25,700 Mm^3 for 2010) are quite similar to ISIMIP's values (39,709 Mm^3 and 27,936 Mm^3), while ISIMIP does not provide an assessment for non-piped WD. However, the spatial detail of our study (2,5 arc minutes or 0.041667 degrees) is incomparable with ISMIP's 0.5 degree. For each of their grid cells, we quantify 144 grid cells, as visible in Supplementary Figure 3. Also, our assessment includes real data from +450 water supply utilities, whereas ISIMIP only downscales national values. Our assessment aggregated to 0.5 degrees (Supplementary Figure 3B), shows the loss in spatial detail. We compare this aggregated version with the spatial data of ISIMIP (Supplementary Figure 3C). On the national level, there is much agreement between the data, with a R^2 of 0.94 and a Median of Differences (MoD) of -27% (Supplementary Figure 3D). On the grid level, the agreement is less, with a R^2 of 0.91 and a MoD of -181%, the latter values showing substantial differences (Supplementary Figure 3D).

Supplementary Table 7: Comparison of our results with ISIMIP[158,159]

**Study/ source**	**Sector**	**Year**	**Spatial resolution**	**Mm^3**	**Open access**	**Our study**
ISIMIP[158,159]	Municipal – only piped access	2020	0.5 degrees, i.e. 50-55 km	39,706	yes	35,807 Mm^3 piped access, 23,989 Mm^3 non-piped access, in resolution 0.041666667 degrees (so for each ISIMIP grid cell we have 144 grid cells)
		2010		27,936		25,700 Mm^3 piped access, 20,749 Mm^3 non-piped access
	Industrial	2020		30,202		28,322 Mm^3 (without hydropower), with manufacturing industry 15,021 Mm^3, electricity 10,053 Mm^3 and mining 3,247 Mm^3
		2010		18,592		18,826 Mm^3 (without hydropower), with manufacturing industry 11,856, electricity 5,006 Mm^3 and mining 1,964Mm^3

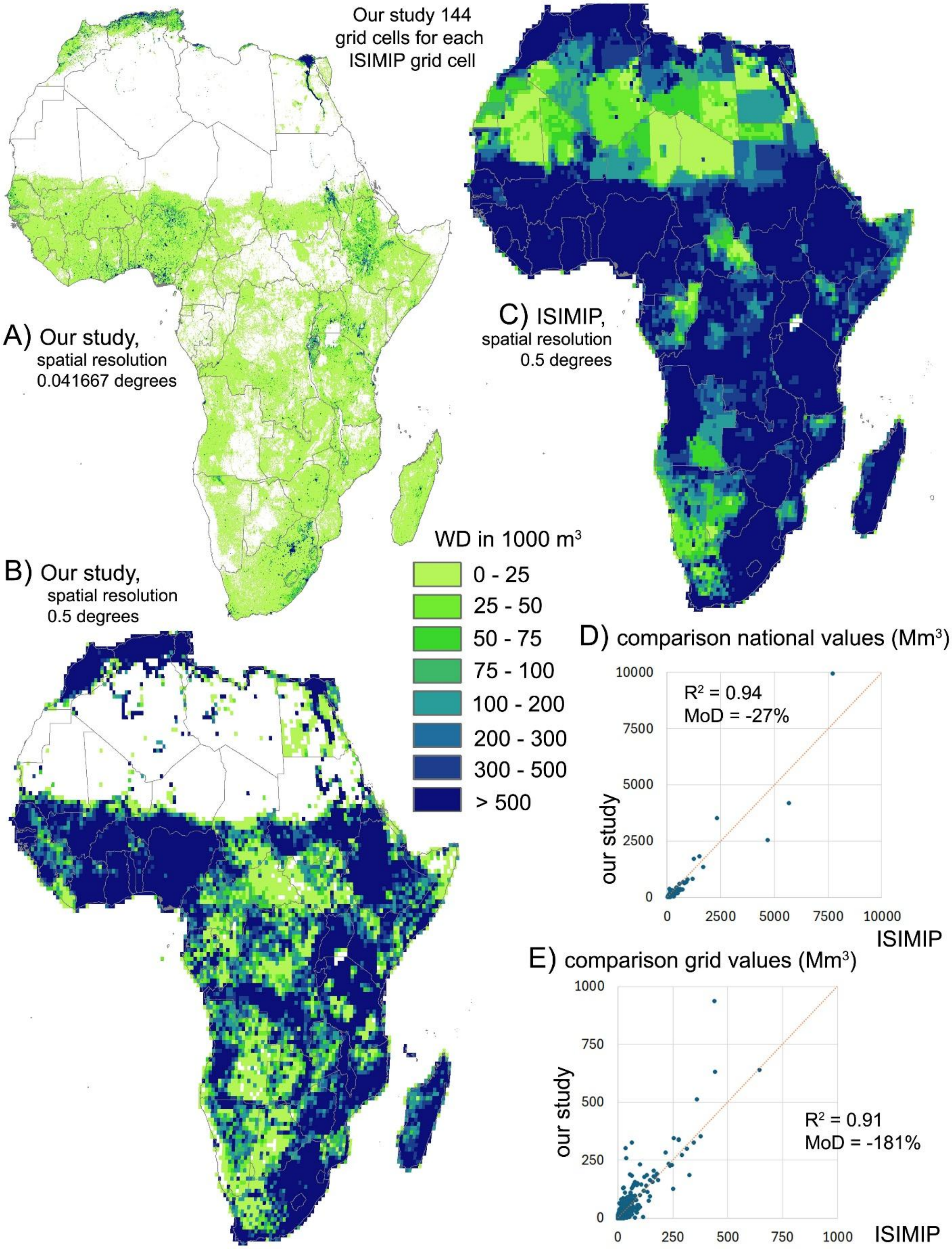

Supplementary Figure 3: Comparison municipal WD (piped access) between our study and ISIMIP for the year 2020. A) Our study in resolution 0.041667 degrees (2.5 arc minute, 5 km); B) our study aggregated to 0.5 degrees; C) ISIMIP; D) comparison national amounts with R^2 spatial correlation and MoD Median of Differences relative to our study; E) comparison grid amounts for spatial resolution 0.5 degrees

Our overall industrial WDs (28,322 Mm^3 for 2020 and 18,826 Mm^3 for 2010) are also quite similar to ISIMIP's values (30,202 Mm^3 and 18,592Mm^3). However, again, the spatial resolution of our assessment is incomparable with ISMIP's 0.5 degree. Also the subsectoral detail of our assessment is far superior to ISMIP's, who only provide overall industry WDs.

Panda and Kim[160] quantified industrial WDs, including manufacturing and thermoelectric water use, for the years 2005, 2010 and 2015 on a global level in a spatial resolution of 0.5 degrees. They published their results in the year 2024 and open access, which provides the opportunity to compare with our results on the national or grid level . We compare their assessment with our year 2010 results (Supplementary Table 8);

Panda and Kim[160] quantify industrial WW using a random forest regression model by combining socioeconomic, climatic, and geographical datasets, while validating their model on Aquastat[9] national data, which are scarce in Africa. For manufacturing industry, Aquastat provides data for many African countries, for electricity only for Zimbabwe. Panda and Kim[160] compute 136,473 Mm^3 for Africa, without differentiating between subsectors (Supplementary Table 8). We compute 18,826 Mm^3, differentiating between manufacturing industry (11,856 Mm^3), electricity (5,006 Mm^3)(without hydropower) and mining (1,964 Mm^3). Panda and Kim's amount is thus much higher than ours. Our value of 18,826 Mm^3 is of the same magnitude as ISIMIP[158,159] (18,592 Mm^3) and Huang et al[12] (13,357 Mm^3), yet lower than Florke et al[13] (36,987 Mm^3, of which manufacturing industry 15,248 Mm^3 and electricity 21,739 Mm^3, Supplementary Table 5). We already described that the estimation of the WD for electricity of Florke et al[13] seems on the high side. The overall global estimation of industrial WD of Panda and Kim (1,133,573 Mm^3) is close to the AQUASTAT amount (905,000 Mm^3), yet for Africa their quantification seems on the high side. They validated their model with Aquastat data, with a specific focus on China and the USA, while acknowledging the data scarcity for Africa.

Panda and Kim[160] consistently estimate higher national total industry WDs as compared to our study (Supplementary Figure 4F). The correlation between our values and theirs is low at R^2 0.43, and the MoD is with +991% very high. All difference values for each country are positive amounts, also visible from the fact that all value stay below the red 1:1 line in the graph. The only country where our study has a low difference is Egypt, where we computed 6,748 Mm^3 and Panda and Kim[160] 7,208 Mm^3 (difference +7%).

Supplementary Table 8: Comparison of our results with Panda and Kim[160]

**Study/ source**	**Sector**	**Year**	**Spatial resolution**	**Mm³**	**Open access**	**Our study**
Panda and Kim[160]	Gridded global dataset of industrial water use, including manufacturing and thermoelectric water use<br>Based on Aquastat[9] national data<br>WRI's Global Power Plant Database[162] was used for downscaling thermoelectric water use, based on power plant production, yet without accounting for different water intensities of fuel types<br>Annual from 2000 to 2015, year 2010 can be compared	2010	0.5°, 50-55 km	136,473, global 1,133,573 (Aquastat 905,000)<br>Example RSA 11,007<br>Example Egypt 7,159	yes	18,826 Mm^3, with manufacturing industry 11,856, electricity 5,006 Mm^3 (without hydropower) and mining 1,964Mm^3<br>Example RSA: 1,892 for manufacturing industry +<br>(without hydropower)<br>Example Egypt 2,470 for manufacturing industry +

In Supplementary Figure 4A to 4C we show the spatial level of detail we provide in our assessment, which is unprecedented on the spatial and subsectoral level and therefore incomparable in level of detail compared to Panda and Kim[160]. For electricity, we account for 1,127 individual non-hydro power plants differentiated according to fuel type. For manufacturing industry, we account for subsectoral industries and have a spatial resolution of 0.0416667 degrees as compared to Panda and Kim's 0.5 degrees. We thus have 144 grid cells for each of their grid cells. For mining, we account for 385 individual mines, differentiated according to commodities. We combined and aggregated our WD results to 0.5 degrees (Supplementary Figure 4D) to compare our results to Panda and Kim (Supplementary Figure 4E).

Comparing on the individual grid level, there is a very poor spatial correlation (R^2=0.02) and the MoD is +30%, as for many grid cells our results vary between lower or higher amounts compared to Panda and Kim. This is also clearly visible by comparing Supplementary Figure 4D and 4E, as the spatial pattern between the two studies differs.

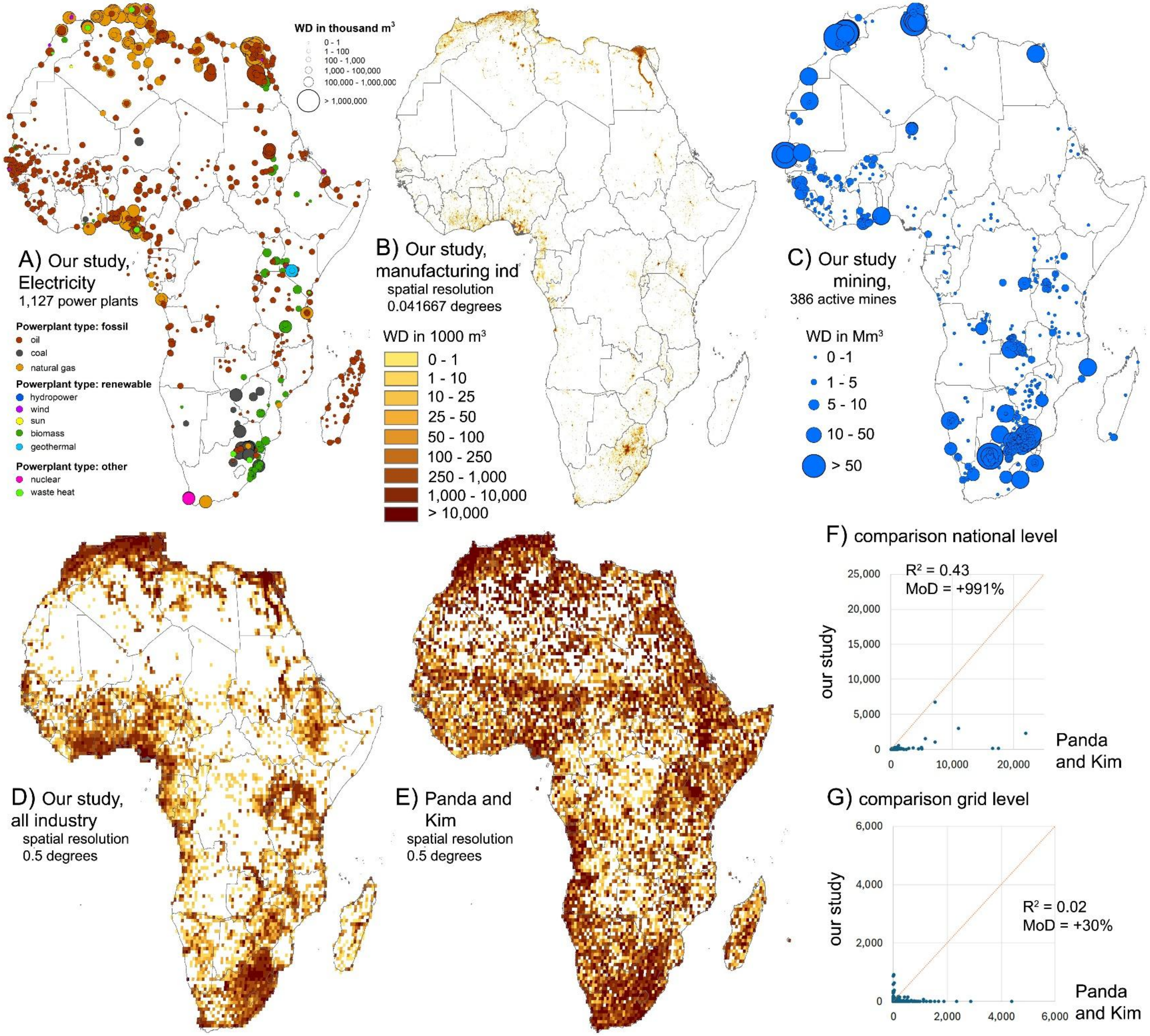

Supplementary Figure 4: Comparison industrial WD between our study and Panda and Kim[160] for the year 2010, A) Our study electricity (non-hydro); B) our study manufacturing industry in resolution 0.041667 degrees ; C) our study mining; D) all three industrial sectors aggregated to all industry resolution 0.5 degrees; E) Panda and Kim 0.5 degrees; F) comparison national amounts (Mm^3) with R^2 spatial correlation and MoD Median of Differences relative to our study; G) comparison grid amounts for spatial resolution 0.5 degrees

Panda and Kim[160] describe that they use WRI's Global Power Plant Database[162] in their random forest regression model. However, both in terms of number of power plants according to fuel type as well as installed capacities accounting, our power plant database provides many more data and spatial detail (Supplementary Table 9). While we account for different water intensities of different fuel types, thereby using a bottom-up approach, Panda and Kim use total power plant production to downscale country-wise industrial water use data to a grid.

Supplementary Table 9: Comparison of our own developed power plant database for the years 2020 and 2010 with WRI's Global Power Plant Database[162]

	This study. The data include the small islands of Africa, in order to compare with other studies				WRI's Global Power Plant Database[162]	
	year 2020		year 2010		year 2020	
	Number	Capacity (MW)	Number	Capacity (MW)	Number	Capacity (MW)
Oil	1,054	22,265	827	11,722	102	8,425
Coal	49	49,807	30	38,343	31	45,097
Natural gas	343	119,263	185	51,430	134	64,293
Hydropower (reservoir + ROR)	561 (183 reservoir +378 ROR)	36,892	396	27,062	163	30,399
Wind	83	5,646	17	908	42	4,499
Sun	299	6,150	7	38	129	4,893
Biomass	121	1,958	74	1,109	15	320
Geo	8	813	3	429	7	761
Nuclear	1	1,860	1	1,860	1	1,800
Waste heat	15	951	7	772	7	46
**total**	**2,534**	**245,604**	**1,553**	**133,674**	**631**	**160,533**

For additional validation of our manufacturing industry WD results, we conducted industrial WD quantifications for selected industrial sub-sectors where outputs are available in weight quantities, by means of Equation 10.

The food and beverage processing industry is a sub-sector where such data are available on the national level, through the FAO Food Balance Sheets (FAO-FBSs)[25]. A FAO-FBS provides a comprehensive picture of the agrifood situation of a country in a specified reference period, showing the pattern of a country's food supply and utilizations. A comparison of the values obtained by means of the FAO-FBSs for the year 2020 (Equation 10) and the values obtained in the original assessment by means of the Gross Value Added (GVA, Equation 8), shows a very high correlation of 0.947 (R^2) and a low Median of Differences (MoD) of 22% (Supplementary Figure 5 and Supplementary Table 10). This means that both methodologies provide similar amounts, validating our original approach.

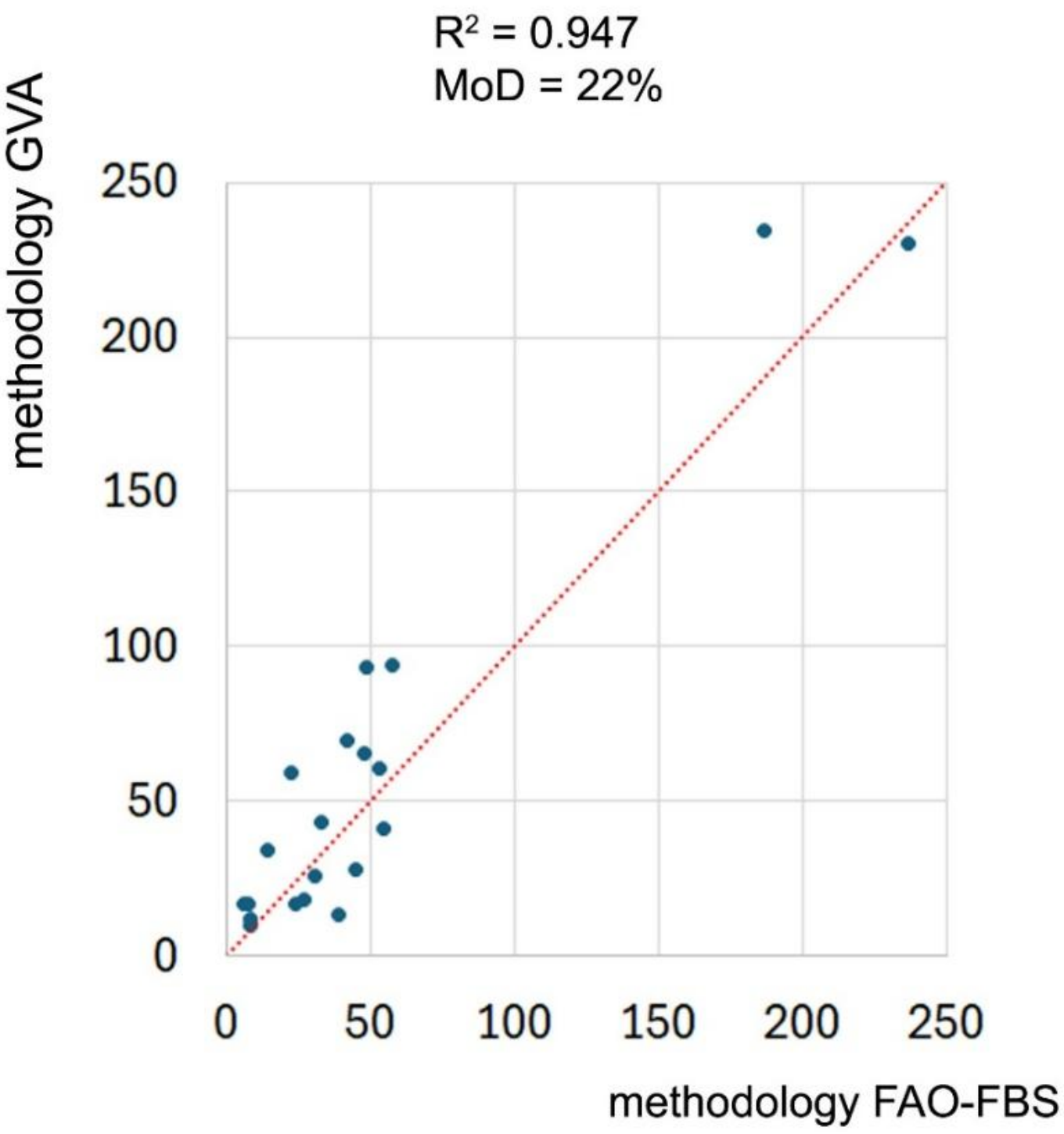

Supplementary Figure 5: Industrial WD amounts for the subsector food and beverages industry in Mm3 computed with the FAO-FBS approach (X-axis) and the GVA approach (Y-axis), year 2020

As an example, countries with higher amounts are South Africa, where the values are 187 Mm^3 for FAO-FBS and 235 Mm^3 for GVA, respectively, whereas for Egypt, the values are 237 Mm^3 and 235 Mm^3. These are very similar amounts which validate our statement that our values are best estimates, recognizing uncertainty.

Supplementary Table 10: Comparison manufacturing industry subsector food and beverages processing WD amounts 2020 between FAO-FBS processing weight calculation (Equation 10) and GVA calculation (Equation 8)

Country	Processing 1000 Tons QUANTITYind, sub-sectorX in Equation 10	Amount (Mm^3) FAO-FBS methodology	Amount (Mm^3) GVA methodology
South Africa	23,367	187	235
Egypt	29,631	237	231
Cameroon	1,191	55	41
Congo	758	6	17
Côte d'Ivoire	5,253	42	70
Eswatini	5,629	45	28
Ethiopia	4,103	33	43
Ghana	2,779	22	59
Kenya	6,094	49	93
Madagascar	945	8	17
Malawi	3,011	24	17
Morocco	5,948	48	65
Niger	1,017	8	10
Rwanda	14,659	9	12
Senegal	1,072	15	34
Tanzania	6,618	53	60
Uganda	7,205	58	94
Tunisia	3,359	27	18
Zambia	4,888	39	14
Zimbabwe	3,883	31	26

We also applied Equation 10 to the subsector "clothing" for Morocco, the only country for which we found reliable weight production data (annual production of 950,000 ton[166]) and for which an average water intensity of 200 m^3 per ton can be assumed[167–170]. This results in a WD of 190 Mm^3, whereas the GVA methodology (Equation 8) results in a WD of 197 Mm^3. Again, these are very similar amounts which validate our statement that our values are best estimates, while recognizing uncertainty.

Applying Equation 10 to the subsector "paper and paper products" for South Africa, with an annual production of 1,891,283 ton for the year 2020[25] and an average water intensity of 70 m^3 per ton[171,172], results in a WD of 132 Mm^3, whereas the GVA methodology (Equation 8) results in a WD of 151 Mm^3 for the year 2020. These are again quite similar amounts which validate our statement that our values are best estimates, while recognizing uncertainty.

4 Uncertainty and sensitivity

We conducted a spatial Monte-Carlo analysis by randomly varying IE (Irrigation Efficiency) amounts between ±20% and their effect on irrigation WDs for the year 2020 (Supplementary Figure 6 and Table 11). The deterministic amount we present in our study is 225,174 Mm^3, which is within the 90% confidence interval (215,151 - 231,399 Mm^3), the 95% confidence interval (213,736 - 232,818 Mm^3) and the 99% confidence interval (211,183 - 235,372 Mm^3). Our deterministic amount is close to the average (223,283 Mm^3) and median (223,285 Mm^3) amount of the Monte Carlo analysis.

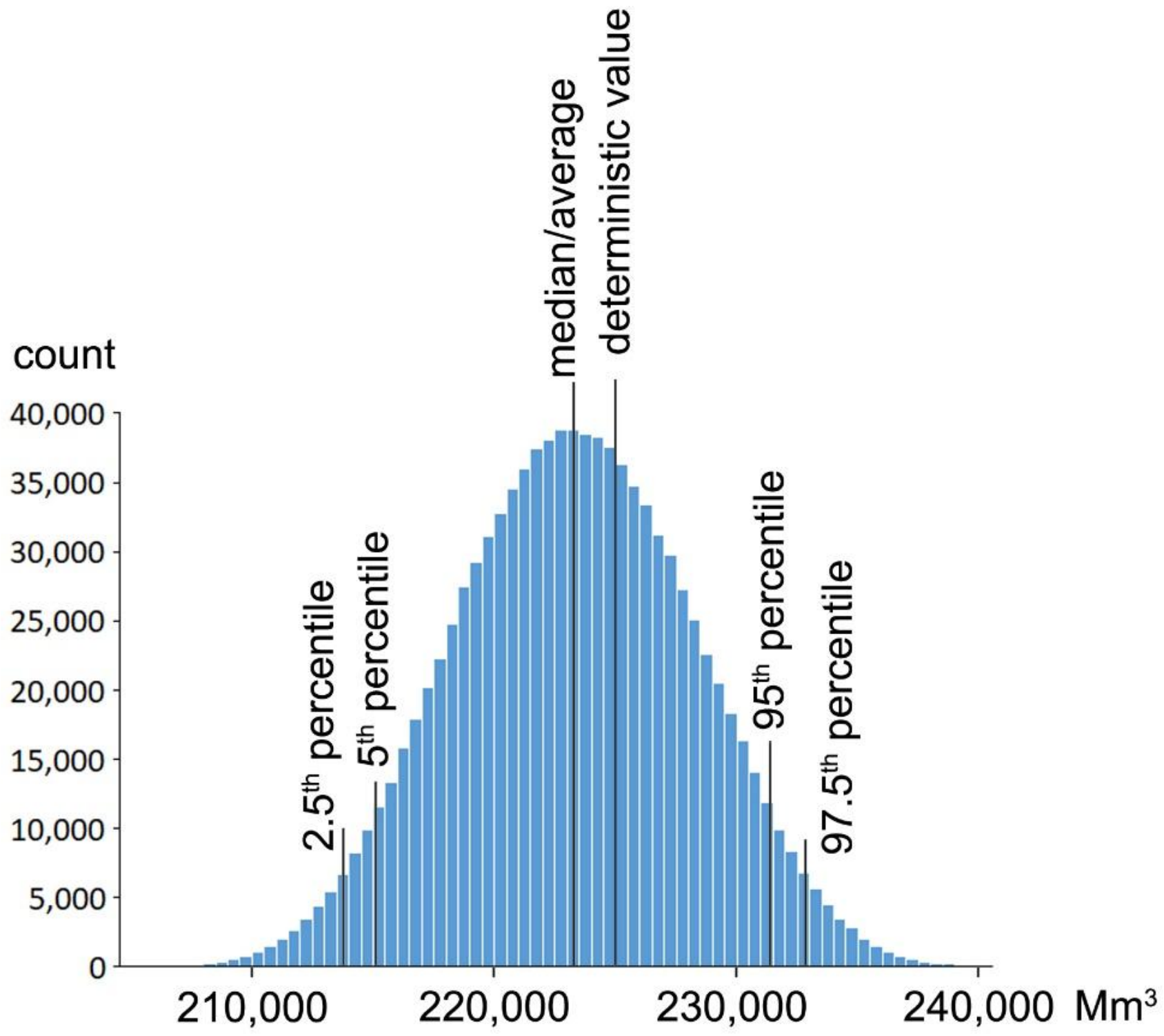

Supplementary Figure 6: Monte-Carlo analysis irrigation WD for the year 2020 (amounts in Mm^3), with irrigation efficiency (IE) varying between ±20% in 1 million iterations

Supplementary Table 11: Monte-Carlo analysis values for Supplementary Figure 6

Description	Amount
Deterministic value	225,174
Mean value	223,283
Standard deviation	4,933
25th percentile	219,838
50th percentile/median	223,285
75th percentile	226,728

**Description**	**Amount**
90% Confidence interval	215,151 (5th percentile) - 231,399 (95 percentile)
95% Confidence interval	213,736 (2.5th percentile) - 232,818 (97.5th percentile)
99% Confidence interval	211,183 (0.5th percentile) - 235,372 (99.5th percentile)

We conduct a sensitivity analysis for municipal WDs in the year 2020, by varying the factors LOSSmunicipal (physical water loss) and FACTORmunicipal (the proportion of domestic water use within municipal water use in %), as used in equations 1 to 4. Decreasing $LOSS_{municipal}$ by -20% results in a reduction of total WD from 59,796 Mm^3 to 56,641 Mm^3 (-5%). Increasing $LOSS_{municipal}$ by +20% results in an increase of total WD from 59,796 Mm^3 to 64,293 Mm^3 (+8%). There is much uncertainty on physical water loss of current water supply utilities, especially when covering the whole of Africa. Yet, as we are the first study to use actual water loss data of +450 utilities continent-wide, our study is a best estimate in municipal WD, unprecedented in the available scientific literature.

Supplementary Table 12: Sensitivity analysis WDmun, by varying $FACTOR_{municipal}$ and $LOSS_{municipal}$ with +/- 20%, analysis conducted for each of the 1.48 million grid cells of the raster in 2,5 arc-minutes resolution

		Total	Piped	Non-piped
Our study	Mm^3	59,796	35,807	23,989
$LOSS_{municipal}$ -20% and $FACTOR_{municipal}$ constant	Mm^3	56,641	34,904	21,737
	% change from original value	-5	-3	-9
$LOSS_{municipal}$ +20% and $FACTOR_{municipal}$ constant	Mm^3	64,293	37,203	27,090
	% change from original value	+8	+4	+13
$LOSS_{municipal}$ constant and 100-$FACTOR_{municipal}$ -20%	Mm^3	57,772	35,176	22,596
	% change from original value	-3	-2	-6
$LOSS_{municipal}$ constant and	Mm^3	62,241	36,610	25,631

		Total	Piped	Non-piped
100-FACTOR$_{municipal}$ +20%	% change from original value	+4	+2	+7

We also vary FACTOR$_{municipal}$ (the proportion of domestic water use within municipal water use in %), by using 100-FACTOR$_{municipal}$ +/- 20%, the latter being typically about 20-30% of total municipal WD. A reduction of -20%, results in municipal WD decreasing from 59,796 Mm^3 to 57,772 Mm^3 (-3%). An increase with +20%, results in municipal WD increasing from 59,796 Mm^3 to 62,241 Mm^3 (+4%). Thes amounts give insight in the sensitivity of our results to specific factors.

We also conduct a sensitivity analysis for our mining WD, which we quantify at 3,247 Mm^3 for the year 2020 (Supplementary Table 13). Using only mine production amounts for the year 2019 results in a WD of 3,398 Mm^3 (+5%), whereas for the year 2021 this results in a WD of 3,759Mm^3 (+16%). This shows the generally lower mine production amounts during the year 2020 due to the COVID pandemic. For these different years individual mine production amounts showed slight variations. When only selecting the minimum annual production amounts for each mine during this three year period, the absolute Africa-wide minimum WD decreases to 3,001 Mm^3 (-8%). When only selecting maximum annual production amounts for each mine, this overall WD increases to 3,952 Mm^3 (+22%). This shows again that our quantifications are to be regarded as best estimates, not as absolute amounts.

Supplementary Table 13: Sensitivity analysis mining WD year 2020

**Description**	**Amount in Mm^3**	**Change amount in Mm^3**	**Change amount in %**
Deterministic value our study year 2020	3,247		
Deterministic value mine production amounts year 2019	3,398	151	+5
Deterministic value mine production amounts year 2021	3,759	512	+16
Deterministic value minimum annual mine production amounts year 2019, 2020 and 2021	3,001	-246	-8
Deterministic value maximum annual mine production amounts year 2019, 2020 and 2021	3,952	705	+22
